# Personalized ctDNA analysis for detection of residual disease and recurrence in surgically treated HNSCC patients

**DOI:** 10.1038/s41698-026-01309-0

**Published:** 2026-02-03

**Authors:** Susanne Flach, Christodoulos Pipinikas, Tom Huberty, Axel Lechner, Clodagh Murray, Giovanni Marsico, Karen Howarth, Christoph Walz, Lukas Käsmann, Andreas Mock, Philipp Jurmeister, Kristian Unger, Sophia Stöcklein, Gizem Abaci, Nitzan Rosenfeld, Christoph A. Reichel, Olivier Gires, Martin Canis, Philipp Baumeister

**Affiliations:** 1 Department of Otorhinolaryngology, Head and Neck Surgery, University Hospital, LMU Munich, Munich, Germany; 2 German Cancer Consortium (DKTK), Partner Site Munich, Germany; 3 Bavarian Cancer Research Center, Munich, Germany; 4 NeoGenomics Inc., Fort Myers, FL USA; 5 The Bradfield Centre, Cambridge, UK; 6 Institute of Pathology, Faculty of Medicine, LMU Munich, Munich, Germany; 7 Department of Radiation Oncology, University Hospital, LMU Munich, Munich, Germany; 8 Research Unit Translational Metabolic Oncology, Institute for Diabetes and Cancer, Helmholtz Zentrum München – German Research Center for Environmental Health (GmbH), Neuherberg, Germany; 9 Department of Radiology, LMU Klinikum, Munich, Germany; 10 Barts Cancer Institute, Queen Mary University of London, London, UK

**Keywords:** Biomarkers, Cancer, Computational biology and bioinformatics, Oncology

## Abstract

Despite advances in multimodal therapy, survival in advanced head and neck squamous cell carcinoma (HNSCC) has improved only modestly. Tumor-informed circulating tumor DNA (ctDNA) assays allow early detection of molecular residual disease (MRD) and recurrence after curative treatment. We analyzed ctDNA in plasma from 76 and saliva from 54 HNSCC patients before and after curative-intent surgery, testing 656 plasma and 128 saliva samples longitudinally. High preoperative ctDNA shedding correlated with advanced pathological stage, lymph node involvement, adverse histologic features, and molecular markers including PD-1 expression and tumor mutational burden. Transcriptomic profiling showed associations between high shedding and increased proliferation, EGFR/MAPK pathway activity, and upregulation of EGFR-related invasion and metastasis genes. Plasma ctDNA detected ≥14 days post-surgery identified 91.3% of recurrences, with lead times up to 500 days before clinical confirmation. These results highlight the value of serial ctDNA monitoring for early relapse detection and potentially improved treatment guidance in HNSCC.

## Introduction

Head and neck squamous cell carcinoma (HNSCC) is a heterogeneous disease. While human papilloma virus (HPV)-positive tumors have a better prognosis, HPV-negative HNSCC remains a devastating disease^1^. Early relapse and distant metastases drive poor outcomes, particularly when recurrence is detected too late for curative-intent local therapy^2^. Treatment requires aggressive multimodal approaches, including surgery with adjuvant (chemo)radiotherapy ((C)RT) or primary CRT – which often cause lasting impairments in breathing, swallowing, and speech^3^.

About 30% of patients with HNSCC develop locoregional recurrences and/or second primary tumors (SPTs), frequently within two years after completion of treatment^4–7^. Five-year overall survival (OS) in these cases is generally poor: 0% to 32% for those receiving CRT and 26% to 67% for patients undergoing salvage surgery, the recommended curative option regardless of recurrence site or stage^8–10^. Early recurrences are often asymptomatic^11^ and clinically undetectable, highlighting the need for timely detection while salvage surgery remains feasible. Overall, survival in patients with recurrence after salvage surgery remains low, with most patients dying within a year of recurrence^12,13^.

Current surveillance methods for detecting recurrences and SPTs lack sufficient evidence of efficacy^14^. Monitoring relies on clinical examinations and imaging, both inconsistently applied due to an absence of clear guidelines and poor patient adherence^15^. Post-treatment anatomical changes—such as trismus, edema, and reconstructive surgery—often impair endoscopic evaluation, leading to frequent reliance on imaging or panendoscopy under general anesthesia.

Sensitive detection of occult disease or recurrence is essential. Circulating cell-free tumor DNA (ctDNA) analysis has shown promise for relapse prediction in several solid tumors^16–43^. While molecular residual disease (MRD) can refer broadly to residual tumor cells, in this context it denotes tumor-derived DNA fragments detected shortly after curative-intent treatment in blood or saliva^42^. Prospective studies using ctDNA to assess MRD in surgically treated HNSCC are limited by small sample sizes^37,39,41,44,45^. Nonetheless, early MRD detection offers a critical window for additional surgery and/or adjuvant (C)RT. As HNSCC recurrences are mainly locoregional, ctDNA-guided monitoring could enable earlier detection and curative intervention, potentially improving clinical outcomes. ctDNA-negative patients, in turn, may avoid unnecessary treatment and diagnostic procedures, therefore potentially reducing associated risks and preserving quality of life.

Here, we report results from LIONESS (**L**iquid B**IO**psy for Mi**N**imal R**ES**idual Di**S**ease Detection in Head and Neck Squamous Cell Carcinoma), a prospective cohort study evaluating tumor-informed ctDNA detection of MRD and molecular relapse prior to clinical recurrence in patients with HNSCC after curative-intent surgery. Findings from the first 17 patients have been published previously^37^. In addition, RNA sequencing (RNA-seq) and immunohistochemistry of primary tumors were performed to investigate clinicopathological and microenvironmental differences between tumors with high ctDNA shedding and those with low or no ctDNA shedding.

## Results

### Patient baseline characteristics

**Fig. 1:**
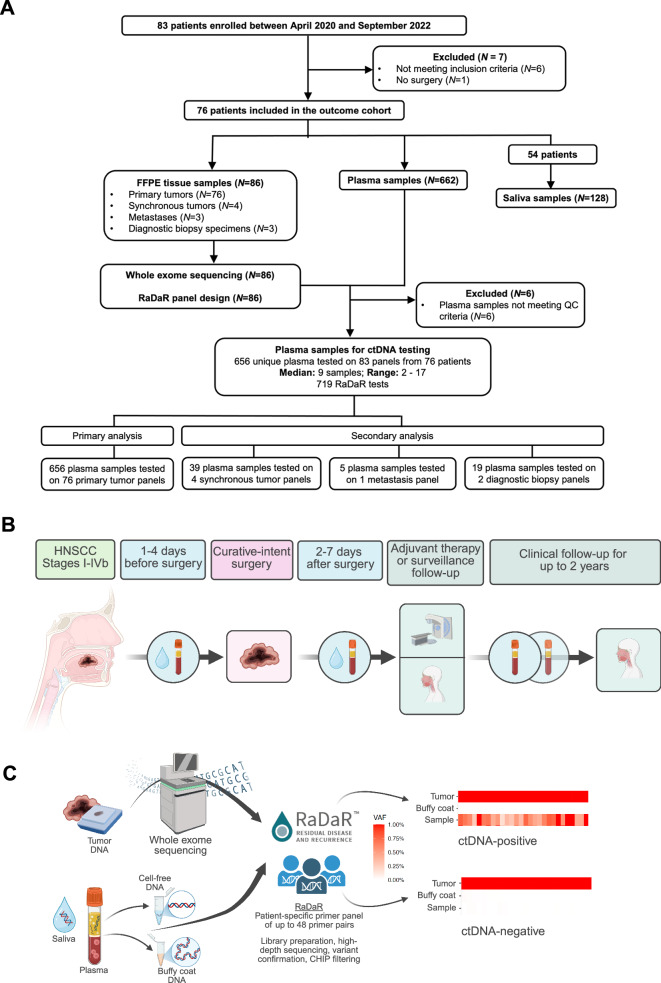
LIONESS study design. **A** Prospective cohort study population flow diagram showing the study population based on available tissue and testing. **B** Schematic of LIONESS study design. **C** RaDaR workflow. Tumor tissue from surgical resection was macrodissected and used for WES to identify somatic mutations followed by designing personalized ctDNA assays. Tumor and buffy coat DNA were analyzed using personalized assays to confirm somatic mutations and exclude germline and clonal hematopoiesis of indeterminate potential (CHIP) variants. Plasma and saliva samples were collected pre- and postoperatively, analyzed using RaDaR panels and high-depth sequencing, and ctDNA detection reported per patient. WES, whole exome sequencing; VAF, variant allele frequency. Figure generated with BioRender.com.

Between April 2020 and September 2022, 83 HNSCC patients were consented and enrolled (Fig. 1A). After excluding six patients (withdrawal of consent, no invasive carcinoma in final specimen, hemolyzed samples) and one patient whose final histopathology revealed an adenosquamous carcinoma, 76 patients were followed up for ctDNA analysis. The median age was 64 years (range: 35 years – 83 years), 80.3% (61/76) of patients were male, and most patients (48/76, 63.2%) had UICC stage III/IV disease and were diagnosed with p16-negative HNSCC (71/76, 93.4%) (Table 1; Supplementary Data 1 – File 1). At inclusion, 3/76 patients presented with recurrent disease and/or a SPT of the upper aerodigestive tract. All participants had blood and saliva samples taken one to four days before surgery, two to seven days after surgery, and during regular clinical follow-up (Fig. 1B).

**Table 1:** Baseline patient characteristics in the LIONESS study cohort

Characteristics	Category	*N* (% total)
Age (years) at diagnosis	Median (range)	64 (35-83)
Sex	Male	61 (80.3)
	Female	15 (19.7)
Smoking history	Current/former	66 (86.8)
	Never	10 (13.2)
Pack years	Median (range)	40 (3-150)
Primary tumor location	Oral cavity	21 (27.6)
	Oropharynx	12 (15.8)
	Hypopharynx	15 (19.7)
	Larynx	28 (36.9)
Pathological T stage	T1	11 (14.5)
	T2	23 (30.2)
	T3	32 (42.1)
	T4	10 (13.2)
Pathological N stage	N0	35 (46.0)
	N1-N3	36 (47.4)
	NX	5 (6.6)
Extranodal extension	Yes	16 (22.5)
	No	55 (77.5)
Lymphatic vessel invasion	L0	56 (73.7)
	L1	20 (26.3)
Vascular invasion	V0	74 (97.4)
	V1	2 (2.6)
Perineural invasion	Pn0	66 (86.8)
	Pn1	10 (13.2)
Grading	G1	3 (4.2)
	G2	47 (66.2)
	G3	20 (28.2)
	G4	1 (1.4)
UICC stage (8th edition)	Stage I	13 (17.1)
	Stage II	15 (19.7)
	Stage III	22 (29.0)
	Stage IVa	15 (19.7)
	Stage IVb	11 (14.5)
p16 status	Positive	5 (6.6)
	Negative	71 (93.4)
Neck dissection	Bilateral	60 (78.9)
	Ipsilateral	11 (14.5)
	None	5 (6.6)
Adjuvant therapy	Radiotherapy	18 (23.6)
	Chemoradiotherapy	29 (38.2)
	None (clinical controls)	29 (38.2)

### RaDaR^®^ quality metrics: WES, panel design, qualification, and plasma profiling

Whole exome sequencing (WES) was performed on formalin-fixed paraffin-embedded (FFPE) tissue from 86 tumor specimens across 76 patients (Supplementary Data 1 – File 2). Two patients had synchronous primary HNSCCs and one patient had a synchronous pT1 renal cell carcinoma. Additionally, a lymph node metastasis, a metachronous primary tumor, and two distant metastases were sequenced. Diagnostic biopsies were analyzed for three patients. RaDaR assays were successfully designed for all patients (median: 48 variants, range: 17-60) to track ctDNA in pre- and postoperative plasma and saliva (Fig. 1C; Supplementary Data 1 – File 3 and File 4). Primary MRD analysis used panels from resected specimens to assess 656 plasma samples (median: 9 per patient, range 2-17). Secondary analyses used panels from SPTs (*N* = 4), diagnostic biopsies (*N* = 2), and metastases (*N* = 1) on 63 plasma samples. 719 RaDaR tests were performed using 83 panels from 76 patients (Fig. 1A).

Overall, ctDNA was detected at any timepoint in 26% of samples (170/656) in 66/76 patients (87%) at a median estimated variant allele frequency (eVAF) of 0.036% (range: 0.00033% - 18.43%) with 35% of samples (59/170) having an eVAF of ≤0.01%.

### ctDNA detection in preoperative plasma samples

**Fig. 2:**
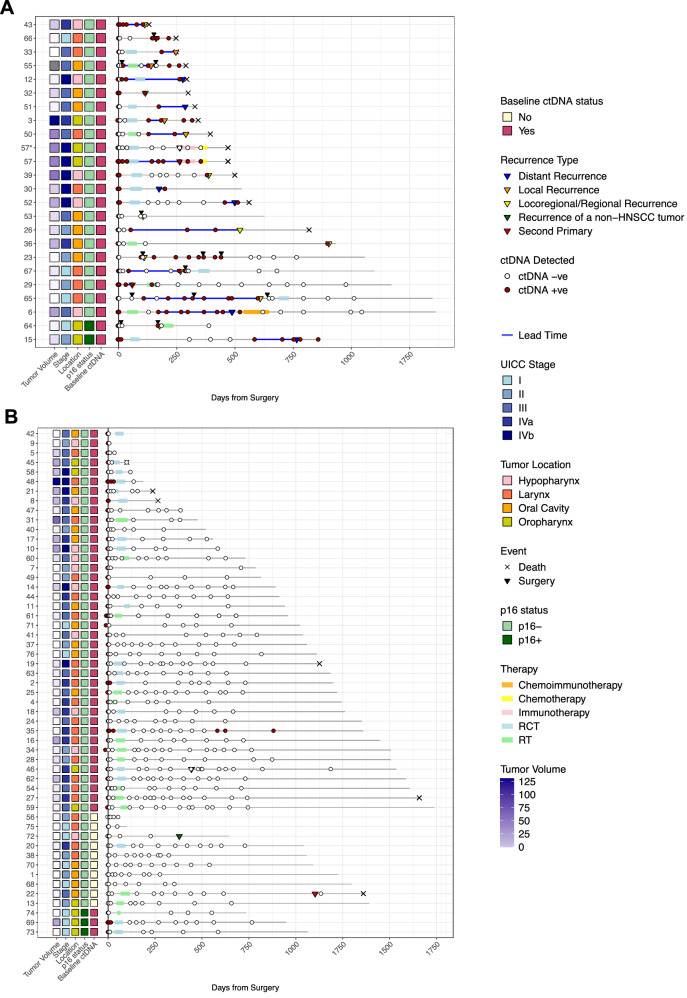
Swimmer plots showing ctDNA status of each sample tested per patient after curative-intent surgery (*N* = 76). Each line represents a specific patient. Longitudinal monitoring of serial plasma samples is shown, indicating when ctDNA was detected (red circle) or not detected (white circle). The black line extends across the x-axis to the last clinical visit to date to indicate the total duration of follow-up for each patient. The solid blue line indicates the lead time which is the interval between the first ctDNA positive sample taken ≥14 days post-surgery and clinical confirmation of disease recurrence (inverted yellow triangle). Patients who did experience recurrent disease (*N* = 23) (**A**) and who did not experience recurrent disease (*N* = 53) (**B**) are represented separately. Patient 57 presented with two synchronous primary tumors located in the oropharynx and hypopharynx, both of which underwent WES and ctDNA was tracked separately for each tumor (shown separately in (**A**)). WES, whole exome sequencing.

Preoperative plasma ctDNA was detected in 66 of 76 patients (87%) (Fig. 2) at a median eVAF of 0.067% (range: 0.0011% - 12.6%). 25% of samples had an eVAF ≤0.01%. Detection rates were 71% for stage I/II (20/28 patients) and 96% for stage III/IV (46/48 patients).

Higher % eVAF correlated with larger tumor volumes from computed tomography (CT) staging scans (*p* = 3.3×10^-7^, R^2^ = 0.32) and higher tumor stage (Supplementary Fig. 1A, B). Detection rates were significantly associated with tumor volume (*p* = 0.0011), pathological tumor stage (*p* = 0.00319), nodal metastasis (*p* = 0.025), combined histopathological risk factors – including extranodal extension, perineural invasion, and lymphatic or vascular invasion (*p* = 0.004), and prognostic stage group (UICC 8th edition, *p* = 0.0456). No significant associations were found with extranodal extension (*p* = 0.193), tumor necrosis (*p* = 0.289), vascularization (*p* = 1), or tumor localization (*p* = 0.746) based on preoperative imaging (Supplementary Fig. 2).

### p16-negative HNSCC cases

RaDaR panels for 71 p16-negative HNSCC patients captured a median of 48 variants (QC-confirmed: median 47, range: 9–49). Preoperative ctDNA was detected in 86% (61/71) at a median eVAF of 0.059% (range: 0.001–12.6%). ctDNA detection rate in stage III/IV was 95.8% (46/48). In stage I/II cases, detection was 65.2% (15/23), mainly in tumors of the oral cavity (*N* = 6) and larynx/hypopharynx (*N* = 9). 8/10 ctDNA-negative cases were stage I/II.

### p16-positive oropharyngeal SCC (OPSCC) cases

p16 immunostaining is a well-established surrogate marker for HPV-associated OPSCC, which exhibits distinct tumor biology and treatment response, warranting a separate staging system in the AJCC 8th edition^46–50^. We included five p16-positive cases (6.6% of the cohort). RaDaR panels were successfully designed for all, with a median of 48 variants (QC-confirmed: median 45, range: 36–46). ctDNA was detected preoperatively in all five patients at a median eVAF of 0.067% (range: 0.0012–11.2%).

### Postoperative detection of MRD and recurrence in plasma

Postoperatively, ctDNA was detected in 103 of 579 of plasma samples (17.8%) from 28 of 76 patients (36.8%) at a median eVAF of 0.026% (range: 0.00033–18.4%); 40.8% (42/103) of ctDNA-positive samples had an eVAF ≤0.01%. In p16-negative cases, postoperative ctDNA was detected in 93 of 543 samples (17%) from 25 of 71 patients (median eVAF: 0.022%, range 0.00033–18.4%). In p16-positive cases, postoperative ctDNA was detected in 10 of 36 samples (28%) from three of five patients (median eVAF: 0.113%, range: 0.0009–1.55%). Both p16-positive patients with confirmed recurrence had ctDNA detected prior to relapse.

Patients were followed for a median of 29.5 months (range: 0–57 months) until study closure (April 2025); four were lost to follow-up (median follow-up of 28 days, range: 3 – 63 days) (Supplementary Data 1 – File 5). 33% of patients had a recurrence (23/76) and/or SPT (2/76) with a median interval of eight months between surgery and histopathological (*N* = 22) or radiological (*N* = 3) confirmation (range: 1–29 months), 60.0% (15/25) within the first year. Overall, 19.7% (15/76) had locoregional and 10.5% (8/76) distant metastatic recurrence (soft tissue, lung, and liver). There was no significant correlation between % eVAF and site of recurrence (Supplementary Fig. 3). SPTs in the upper aerodigestive tract were observed in two patients (patients 22 and 46) and remained undetectable by tumor-specific RaDaR panels designed for the primary HNSCC.

**Fig. 3:**
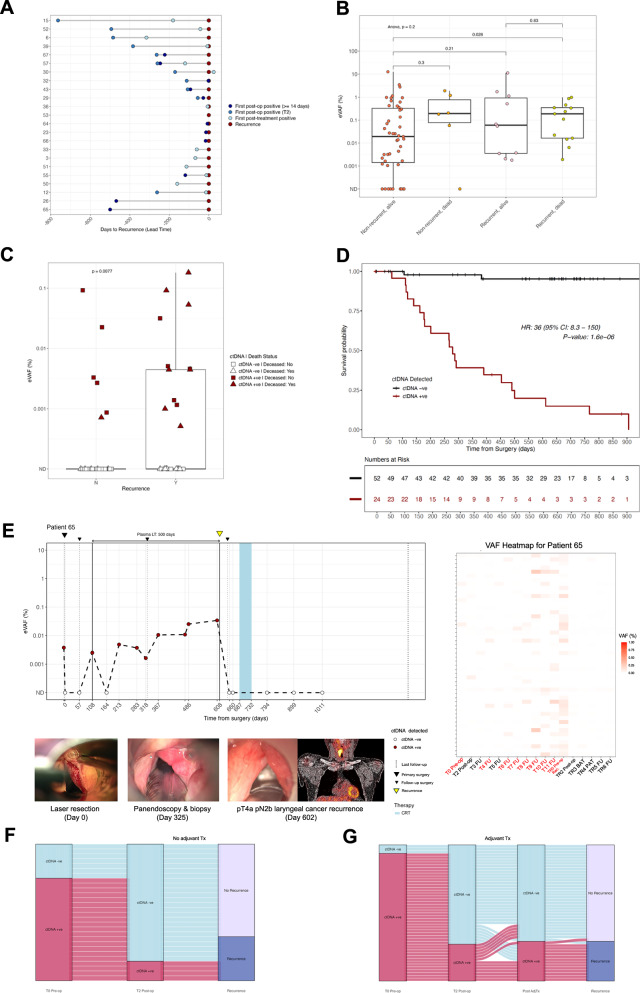
ctDNA analysis in plasma to monitor for MRD and recurrence. **A** MRD detection preceded clinical confirmation of recurrence in 21/23 patients with varying lead times. The first ctDNA-positive sample post-surgery (blue circle), at ≥14 days post-surgery (navy blue circle), and after completion of therapy (light blue circle) prior to confirmation of clinical recurrence for each patient are shown. **B** Results of ANOVA testing including % eVAF ctDNA in preoperative plasma samples, recurrence and whether the patient was alive at that time. Boxplot center line indicates the median, box limits indicate the upper and lower quartiles, and whiskers indicate 1.5x the interquartile range. **C** Boxplot comparing patients with and without disease recurrence based on the % eVAF of ctDNA detection from samples taken two to seven days post-surgery. “Y“ indicating recurrence, “N“ indicating no recurrence. **D** Kaplan-Meier curve of RFS in ctDNA-positive (red) and ctDNA-negative (black) patients post-treatment, including adjuvant therapy, if indicated (*N* = 74). Two patients did not have a post-treatment sample available. P-values were calculated using log-rank tests. **E** Longitudinal plot showing ctDNA changes over time in patient 65. The patient had a pT1b cN0 laser-resected laryngeal cancer and became ctDNA-positive with an eVAF of 0.0025% at day 108 from surgery. Of the subsequent eight samples tested during clinical follow-up, seven were found to be ctDNA-positive albeit at low ctDNA levels. The patient had two control panendoscopies under general anesthesia with negative biopsies at months two and 11, and a CT scan reported as negative for recurrence at month 13 from initial diagnosis. A pT4a pN2b locoregional recurrence was eventually confirmed by a third panendoscopy when random biopsies were taken. A PET/CT scan at this time point showed a submucosal recurrence. In this patient, ctDNA analysis indicated recurrence 500 days prior to histological confirmation. Images of the larynx taken intraoperatively as well as a PET/CT scan at time of recurrence are shown below. A heatmap shows % eVAF of tracked variants over time. **F** Sankey plot of postoperative ctDNA clearance/persistence and recurrence outcome for the 27 patients who did not receive adjuvant therapy. **G** Sankey plot of post-treatment ctDNA clearance/persistence and recurrence outcome for the 49 patients who received adjuvant therapy. MRD molecular residual disease, CT computed tomography, PET positron emission tomography, eVAF estimated variant allele frequency, RFS recurrence-free survival, HR hazard ratio, LT lead time, CRT chemoradiotherapy, FU follow-uo, BAT before adjuvant therapy, PAT post-adjuvant therapy.

ctDNA was detected before clinical relapse in 21 of 23 recurrent cases (91.3% sensitivity), in 88% (7/8) of distant metastases, and 93% (14/15) of locoregional recurrences. Median lead time (LT) was 119 days (range: 14 days–500 days) when considering any ctDNA-positive samples collected ≥14 days post-surgery (median collection interval: 129 days, range: 14 days–897 days) (Fig. 2A and Fig. 3A). Of the two missed p16-negative cases (Supplementary Fig. 4), patient 53 had ctDNA detected (2/48 variants) 10 days before resection of an 8 mm nodal metastasis, but below the positivity threshold. For patient 30, limited pre-relapse sampling (recurrence confirmed 174 days post-surgery) precluded LT determination; ctDNA was detected 199 days post-surgery (Supplementary Fig. 4). More frequent sampling might have enabled earlier molecular relapse detection, increasing sensitivity to 95.6%. Four patients (patients 32, 36, 39, and 64; Supplementary Fig. 4) had a short LT (2–7 days), in three of four cases (patients 32, 36, and 64) due to sparse sampling. Median time from last ctDNA-negative to -positive sample considered in LT calculations was 73 days (range: 8–782 days) (Supplementary Data 1 – File 6). Overall, the assay had 94.2% specificity, with positive and negative predictive values (PPV and NPV) of 87.5% and 96.1%, respectively, when considering ctDNA detection in samples taken ≥14-days from surgery (Supplementary Data 1 – File 7).

Preoperative % eVAF correlated significantly with recurrence and death (*p* = 0.028) (Fig. 3B), however, multivariate analysis was non-significant (*p* = 0.2, ANOVA). Immediate postoperative ctDNA positivity was the main independent prognostic factor associated with a high risk of recurrence (HR = 9.28, 95% CI: 1.66 – 51.86; *p* < 0.01) (Supplementary Fig. 5A; Fig. 3C). The strongest prognostic factor was post-treatment ctDNA positivity, which was associated with a markedly increased risk of recurrence-free survival (RFS) events. In univariable Cox analysis, post-treatment ctDNA detection conferred a 36-fold increased risk of recurrence (HR = 36; 95% CI: 8.3–150; *p* = 1.6 × 10⁻ ^6^) (Fig. 3D), consistent with non-parametric log-rank testing (χ² = 56.8, *p* = 5 × 10^-14^). In multivariable analysis adjusting for clinicopathological covariates, post-treatment ctDNA positivity remained the dominant independent prognostic factor (HR = 237, 95% CI: 18.6–3036; *p* < 0.001). Higher UICC stage also predicted worse outcome, whereas adjuvant therapy appeared to be protective. Other pathological features (margin status, ENE, perineural/vascular/lymphatic invasion) showed trends but were not statistically significant (Supplementary Fig. 5B). This indicates that ctDNA status after completion of treatment is strongly correlated with clinical outcomes. A landmark timepoint could not be defined due to non-uniform sampling.

### Postoperative MRD detection and risk of recurrence during follow-up

In 11 of 23 patients (48%) with recurrence, ctDNA was detected within four weeks from surgery – when adjuvant therapy is still feasible. Four postoperatively ctDNA-positive patients who relapsed did not receive adjuvant therapy due to prior radiotherapy treatment (patient 43), patient choice (patient 32), or tumor board decision (patients 29 and 67) (Supplementary Fig. 4). Two of these (patients 29 and 67) developed local recurrences but remained ctDNA-negative after curative treatment by surgery or definitive CRT. This suggests that ctDNA-positive patients without adjuvant therapy are at higher relapse risk (Fig. 3F), and early intervention may improve outcomes.

Four postoperatively ctDNA-negative patients (patients 23, 26, 65, and 66) who did not receive adjuvant therapy became ctDNA-positive during follow-up prior to relapse (Supplementary Fig. 4). Patient 65 had ctDNA detected with an eVAF of 0.0025% 108 days post-surgery, with low levels persisting (five samples had eVAF of ≤ 0.01%) before a locoregional recurrence was confirmed 500 days later (Fig. 3E).

Regular follow-up is essential due to high long-term risk of SPTs^51,52^, with a 10-year cumulative incidence of 21.8% in laryngeal cancer survivors and 19.5% in survivors of oropharyngeal/oral cavity cancer^7^. SPTs also remain a major cause of mortality among HNSCC survivors^53,54^. Patient 46 developed a genetically distinct SPT in the floor of the mouth one-year post-treatment (Supplementary Fig. 6). The panel designed for the primary tumor failed to detect it, but ctDNA at an eVAF of 0.0082% was found when using a panel based on the SPT itself, albeit only shortly before surgery – likely due to low ctDNA shedding from that site.

Among 53 patients without relapse or SPT (Supplementary Fig. 6), ctDNA was detected postoperatively in six (11.7%). Four patients (patients 2, 14, 48, and 69) showed complete ctDNA clearance after adjuvant therapy, indicating curative potential for MRD-positive disease (Fig. 3G and Supplementary Fig. 6). Of the two remaining patients, patient 8 did not have a post-adjuvant sample, while patient 35 exhibited ctDNA clearance post-CRT, followed by re-emergence >16 months later, yet remained relapse-free throughout a total follow-up of 45 months until study closure (Supplementary Fig. 7). While adjuvant therapy can be curative for some patients with MRD, tumor-specific intrinsic factors—still not fully understood—may drive resistance to CRT and may contribute to later disease relapse^55,56^.

### ctDNA detection in cases with distant metastases

**Fig. 4:**
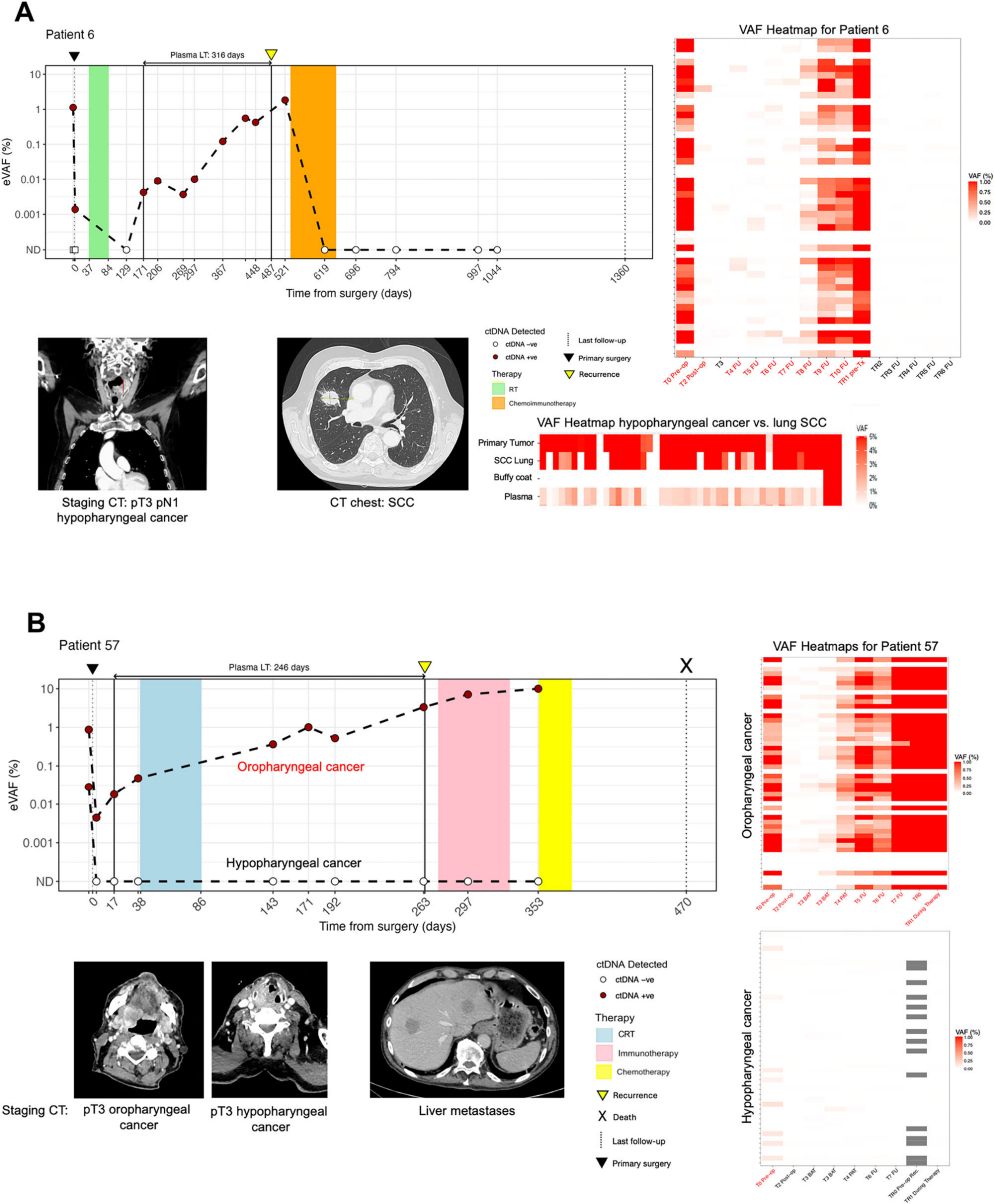
Longitudinal ctDNA profiles of two MRD-positive patients. Longitudinal plot showing ctDNA changes over time in patient 6 (**A**) and patient 57 (**B**). The red circles indicate positive for ctDNA detection while the white circles indicate negative for ctDNA detection. A heatmap shows % eVAF of tracked variants over time for each primary tumor. **A** patient 6 received a total laryngectomy and bilateral neck dissection for a pT3 pN1 hypopharyngeal cancer (CT staging scan shown bottom left) and was ctDNA-positive postoperatively with an eVAF of 0.0014%. Following adjuvant RT, ctDNA was not detected but re-emerged three months after completion of treatment with an eVAF of 0.0043%. ctDNA continued to rise until a CT scan nine months later showed a 2.6 cm×3.2 cm lesion in the right upper lobe of the lung that was histopathologically confirmed as a SCC. Due to inconclusive immunohistochemical analyses, additional DNA methylation profiling was performed, initially suggesting a second primary NSCLC. However, analysis of the DNA from the lung lesion with the personalized panel specific for variants from the patient’s previous hypopharyngeal cancer revealed near-identical genomic profiles, suggesting that the lung lesion was indeed a metastasis rather than a lung SPT (heatmap, bottom right). The patient subsequently started chemotherapy (carboplatin and paclitaxel) and concomitant immunotherapy (pembrolizumab) due to disease progression at that point. Following switch of therapy, all samples tested were ctDNA-negative, pointing to ctDNA clearance in response to this treatment to date. **B** patient 57, diagnosed with two synchronous pT3 pN3b primary tumors in the oropharynx and hypopharynx (CT staging scans shown bottom left) was treated with a total laryngectomy, partial pharyngoglossectomy and bilateral neck dissection. Two personalized panels were designed based on the mutational profiles of the two genetically distinct primary tumors and ctDNA was tracked separately for each tumor. In contrast to the hypopharyngeal tumor where ctDNA remained undetected postoperatively, plasma % eVAF levels continued to increase post-surgery and throughout adjuvant CRT when tested on the panel designed for the oropharyngeal tumor (median: 0.0523%, range: 0.0045–18.43%). A CT scan five months following completion of treatment showed multiple hepatic lesions that were histologically confirmed as SCC metastases. DNA sequencing of a hepatic metastasis confirmed its origin from the oropharyngeal tumor through in silico analysis, which demonstrated high genetic similarity between the two. eVAF estimated variant allele frequency, CT computed tomography, RT radiotherapy, SCC squamous cell carcinoma, NSCLC non-small cell lung cancer, SPT second primary tumor, CRT chemoradiotherapy.

Distant metastases occur in 20–30% of patients after first-line treatment and significantly impact prognosis^57,58^. In our cohort, patient 6 became ctDNA-negative after surgery but ctDNA re-emerged three months after completion of adjuvant RT and rose steadily until a lung SCC was confirmed nine months post-RT. DNA methylation testing initially suggested a second primary NSCLC^59^, but personalized panel analysis confirmed the lesion as a metastasis from the original tumor (Fig. 4A). After starting chemotherapy (carboplatin and paclitaxel) and immunotherapy (pembrolizumab), ctDNA could not be detected, with a PET/CT scan 23 months later showing a non-metabolic residual pulmonary lesion.

Analysis of DNA from metastatic lesions with the previously designed RaDaR assay was also performed for two additional patients with conflicting clinical reports: in patient 52, pleural metastases were traced to originate from a previous hypopharyngeal cancer rather than the initially suspected mesothelioma; in patient 72, liver metastases most likely originated from a prior esophageal SCC, not the hypopharyngeal cancer resected in our clinic.

Postoperative ctDNA monitoring also identified distant metastasis in patient 57, who had synchronous oropharyngeal and hypopharyngeal tumors. Separate RaDaR panels were used for each tumor. While ctDNA from the hypopharyngeal tumor remained undetected after surgery, ctDNA from the oropharyngeal tumor was detected postoperatively and increased during and after CRT (median eVAF: 0.052%, range: 0.0045–18.4%). Liver metastases were later confirmed to originate from the oropharyngeal tumor, indicating possible pre-existing distant disease missed by preoperative imaging (Fig. 4B).

### ctDNA detection in pre- and postoperative saliva samples

In total, 128 saliva or mouth gargle samples were collected from 54 patients. ctDNA was detected in 55% (70/128), with 70% (89/128) concordance with matched plasma (Supplementary Data 1 – File 8). Preoperatively, ctDNA was found in 82% (50/61) of saliva samples across 44 patients (median eVAF: 0.193%, range: 0.00026–13.7%), with highest detection in oral cavity tumors (100%, 21/21) and oropharyngeal tumors (90%, 9/10). Preoperative plasma-saliva concordance was 74% (45/61 samples tested from 40/54 patients), overall highest in oropharyngeal (90%, 9/10) and oral cavity tumors (76%, 16/21) (Supplementary Fig. 8; Supplementary Data 1 – File 9). In six patients (patients 1, 22, 43, 56, 68, and 70) with preoperative ctDNA-negative plasma samples, ctDNA was detected in saliva – five had pT1/pT2 oral cavity tumors. Overall, combined baseline sensitivity across plasma and saliva was 96% (52/54).

Postoperatively, plasma-saliva concordance was 61% (30/49 samples tested from 28/45 patients), with 34.7% of saliva samples (17/49) testing ctDNA-positive (median eVAF: 0.152%, range: 0.00026–13.7%), representing 33.3% of patients. Among 16 patients with confirmed relapse and available postoperative saliva, six (38%) were ctDNA-positive in saliva. In four cases, saliva-based detection improved LT (median 149 days vs. 68 days for plasma) and enabled MRD detection in one plasma ctDNA-negative case. 75% of these tumors were in the oral cavity or oropharynx.

### Comparison of diagnostic biopsy with the resected tumor specimen

Compared to tumor-agnostic approaches, tumor-informed ctDNA assays require more time, which may delay postoperative results critical for adjuvant therapy decision-making. To assess whether preoperative biopsies are suitable for assay design, biopsies and resected tumors from three patients (patients 16, 40, and 61) underwent WES and separate RaDaR panels were created. In two patients, plasma tested with both panels showed almost identical ctDNA profiles and % eVAF levels, indicating preoperative biopsies can reliably inform assay design when WES data is sufficient (Supplementary Data 1 – File 10). For patient 40, an in-silico comparison confirmed similar findings.

### Mutational analysis from WES

**Fig. 5:**
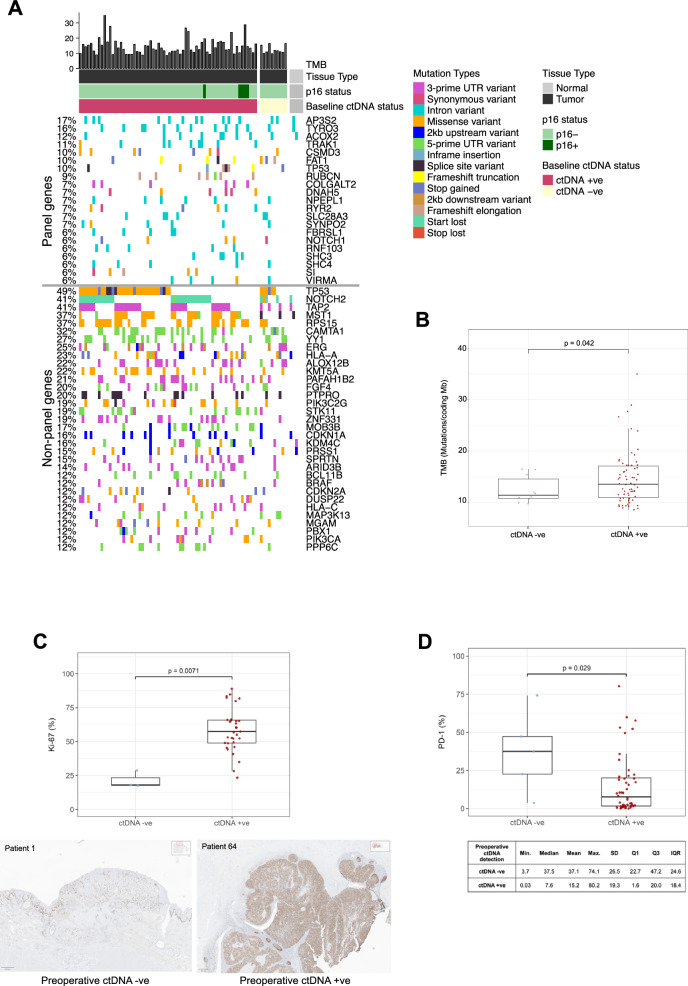
Comparison of mutational and immunohistochemical characteristics between ctDNA high-shedders/low-shedders. **A** The upper panel of the oncoplot illustrates the distribution and types of mutations identified across the top 21 genes used for personalized panel design (*N* = 76). The lower panel highlights the top 33 cancer-related genes exclusively identified in the primary tumors, along with their respective mutation types. **B** Boxplot comparing patients with and without preoperative ctDNA-detection based on TMB. P-value from Welch Two Sample t-test. **C** Boxplot comparing patients with and without preoperative ctDNA-detection based on the Ki67 proliferation index. Shown below are the Ki67 immunohistochemical stainings of a ctDNA non/low-shedder tumor specimen (patient 1) and a ctDNA high-shedder tumor specimen (patient 64). P-value from Wilcoxon test. **D** Boxplot comparing patients with and without preoperative ctDNA-detection based on PD-1 expression. P-value from Mann-Whitney U test. Boxplot center line indicates the median, box limits indicate the upper and lower quartiles. TMB, tumor mutational burden.

Tumor DNA WES was performed at a median target coverage of 278x (range: 129x – 394x), which identified a median of 12.37 mutations per coding megabase (Mb) (range, 7.37 mutations/Mb to 35.02 mutations/Mb). Known mutation types in cancer-related genes based on prior mutational landscapes from the OncoKB list were analyzed. As expected, few recurrent mutations were detected, supporting the use of the tumor-specific panels designed for this study. A search expansion to include panel-specific and non-classical mutations to capture additional relevant alterations showed variants that included intronic changes (*AP3S2*, 17%; *TYRO3*, 16%) and known HNSCC genes (*TP53*, 10%; *FAT1*, 10%; *NOTCH1*, 6%). Mutations in established HNSCC-associated genes, including *NOTCH2* (41%), *CDKN2A* (12%), and *PIK3CA* (12%), were observed exclusively in primary tumors (Fig. 5A). Tumors that were baseline ctDNA-positive (high-shedders, *N* = 66) had a significantly higher tumor mutational burden (TMB) in tumor tissue (median TMB: 13.5 mut./coding Mb, range: 8.56 mut./coding Mb - 35.02 mut./coding Mb) than baseline ctDNA-negative (low- or non-shedders, *N* = 10) tumors (median TMB: 11.4 mut./coding Mb, range: 9.72 mut./coding Mb – 16.46 mut./coding Mb) (p = 0.0424, Welch Two Sample t-test) (Fig. 5B).

### Tumor tissue immunohistochemistry on p16-negative HNSCC

To investigate additional factors influencing ctDNA shedding, immunohistochemical analysis was performed on a subset of primary tumor samples for CD3 and CD8 (*N* = 49), programmed cell death 1 (PD-1) (*N* = 49), programmed cell death 1 ligand 1 (PD-L1) (*N* = 64), and the proliferation marker Ki67 (*N* = 32). Due to the small sample size and different tumor biology p16-positive cases were not included. A significant correlation between ctDNA high-shedding tumors and high Ki67 proliferation index (*p* = 0.007) as well as expression of PD-1 (*p* = 0.029) compared to ctDNA low-shedding tumors (Fig. 5C, D) was identified. No significant associations were observed between the level of ctDNA shedding and CD3/CD8 immunoscores (*p* = 0.189 (I0-I4); *p* = 0.26 for low immunoscore (I0-I1) vs. high immunoscore (I2-I4); Supplementary Fig. 9A) or combined positive scores (PD-L1, p = 0.61; Supplementary Fig. 9B).

### Gene expression pathway analysis in ctDNA high-shedders vs. low/non-shedders in p16-negative HNSCC

ctDNA levels may vary by tumor type^60^ and correlate with tumor volume^61^, though the mechanisms of ctDNA shedding remain unclear. 3’-RNA-seq was performed on intraoperatively collected biopsies from 63 p16-negative tumors; after QC, 58 samples, (49 ctDNA-positive “high-shedders” and nine ctDNA-negative “low/non-shedders” at baseline) were analyzed. Gene Set Enrichment Analysis (GSEA) using the molecular signature database (MSigDB) Hallmarks gene sets revealed enrichment of proliferation-related pathways (“E2F targets”, “MYC targets V1 and V2”, and “G2M checkpoint”) and downregulation of “KRAS signaling” and “TNFα signaling via NFκB” in high-shedders (Supplementary Fig. 10A). High *E2F1* activity further supported a proliferative phenotype (Supplementary Fig. 10B). The most differentially expressed genes of the “*E2F1* targets” in high-shedders vs. low/non-shedders included cyclin dependent kinase inhibitor 2 A (*CDKN2A*), cell division cycle-associated protein 7 (*CDCA7*), and minichromosome maintenance complex component 7 (*MCM7*) (Supplementary Fig. 10C). Inference of signaling pathway activities revealed increased EGFR and MAPK activities and down-regulation of NFκB and TNFα signaling in high-shedders (Supplementary Fig. 10D). Genes driving the EGFR pathway upregulation in high-shedders included cyclin D1 (*CCND1*), amphiregulin (*AREG*), and laminin subunit gamma 2 (*LAMC2*) (Supplementary Fig. 10E). VIPER analysis identified elevated protein activities of heat shock factor 1 (HSF1), metastasis associated 1 (MTA1), and interleukin enhancer-binding factor 3 (ILF3) (Supplementary Fig. 10F), linking proliferation and metastasis-associated pathways to ctDNA shedding.

## Discussion

ctDNA analysis shows promise for detecting MRD and monitoring recurrence after curative-intent surgery^17,25,26,37,62,63^. While ctDNA presence may indicate aggressive tumor biology or metastasis^64^, its prognostic value for survival in patients with HNSCC remains uncertain. Due to the tendency of HNSCC for locoregional recurrence within two years, regular ctDNA monitoring could support early MRD detection and timely adjuvant or salvage therapy. Three recent studies highlight the clinical utility of personalized ctDNA analysis for monitoring MRD in HNSCC. Hanna et al. ^39^. showed that bespoke ctDNA assays sensitively tracked recurrence and treatment response in a heterogeneous cohort of 116 predominantly HPV-negative patients with a median follow-up of 5.1 months. However, most patients received either definitive CRT (46%) or upfront systemic therapy (18%); 17% had distant metastasis at initial staging and 18% of tumors tested p16-positive. Among 100 patients with baseline ctDNA analysis, 75% had ctDNA detected; of the 38 who underwent surgery ± adjuvant (C)RT, ctDNA was detected in 65.8%. Similarly, using a tumor-informed ctDNA assay, Honoré et al.^41^. found that ctDNA positivity within 12 weeks after curative-intent treatment was associated with worse RFS and OS in a cohort of 43 patients, including 26 with stage III/IV p16-negative HNSCC. However, only four patients underwent primary surgery, and ctDNA was detected in just three of the 11 who relapsed, highlighting limitations in detection sensitivity within this small, predominantly non-surgical cohort. Sim et al.^44^. demonstrated that ultrasensitive, whole-genome, tumor-informed ctDNA analysis detected MRD in the first sample taken one to three days after surgery with high sensitivity (86.7%) and specificity (88.9%) for predicting recurrence or death in a predominantly HPV-negative cohort of 24 surgically treated patients with HNSCC. All patients had baseline ctDNA detected; however, the predominance of stage IV disease (75%) limits the generalizability of these findings to early-stage settings.

In our prospective observational cohort study, we analyzed serial pre- and postoperative plasma and saliva samples from 76 patients with stage I–IVb HNSCC using the RaDaR panel to assess MRD and molecular-level recurrence during clinical follow-up. To our knowledge, this represents the largest ctDNA analysis in surgically treated HNSCC to date that combines a comprehensive analysis of ctDNA detection in plasma and saliva with DNA-/RNA-seq and immunohistochemistry of matching primary tumors. We detected baseline plasma ctDNA in 87% of patients, with sensitivity increasing to 96% when saliva was included – emphasizing the value of non-blood sources depending on tumor location^65^. Among patients with early UICC stage I/II disease, 72.5% had baseline ctDNA detected, underscoring the need for highly sensitive assays to capture low ctDNA levels. Compared with Sim et al.^44^, ctDNA was detected in our cohort within two to seven days postoperatively, with an overall sensitivity of 95.7% and specificity of 88.7%.

The amount of circulating ctDNA varies widely – even among patients with similar tumor burden^61,66^ – due to poorly understood biological factors, including differences in cell death mechanisms, cfDNA clearance, and intraday variability^67–69^. This variability may impact the reliability of ctDNA-based assessments and underscores the need for further mechanistic insights. We found that ctDNA shedding correlated with tumor volume, pathological tumor stage, nodal involvement, histopathological risk features, as well as with molecular markers, such as PD-1 expression and TMB. High TMB has previously been shown to correlate with better response to immune checkpoint inhibitors in patients with HNSCC^70^. Transcriptomic profiling revealed that high ctDNA shedding was associated with increased proliferation (Ki67), EGFR/MAPK signaling activity, and upregulation of EGFR pathway signature genes including *CCND1*, *AREG*, *ITGA2*, and *LAMC2* – all linked to invasion and metastasis in HNSCC^71–78^. Overexpression of *AREG* and *ITGA2* has been shown in several solid tumors to be involved in maintenance of oncogenic and metastatic properties^79^ as well as cell adhesion and cell surface-mediated signal transduction^80^. AREG is also a key ligand regulating EGFR signaling strength in newly identified EGFR-activity subtypes of HNSCC, which are linked to epithelial-to-mesenchymal transition (EMT) and local invasion^81^. In addition, *LAMC2*, a marker of partial EMT in HNSCC, has previously been associated with tumor budding, nodal metastasis, perineural invasion, and higher tumor grade in HNSCC^81–83^. *LAMC2* encodes the γ-subunit of Laminin-5, an extracellular matrix component and ligand of integrin β4 (ITGB4), which promotes EGFR-mediated local invasion^81,84^. These data suggest that ctDNA shedding reflects not only tumor burden but also biological aggressiveness. A recent study by Andersen et al.^85^ exploring the biology of ctDNA release in colorectal cancer has also identified tumor size and cell proliferation as key factors of ctDNA shedding.

Postoperative ctDNA detection within four weeks of surgery was strongly associated with recurrence risk; all ctDNA-positive patients who did not receive adjuvant therapy relapsed. These findings align with data from other tumor types^23,24,28,29,33,86,87^ and suggest ctDNA-guided adjuvant therapy may enable escalation in molecularly high-risk patients and future de-escalation in ctDNA-negative cases. However, this approach requires validation in prospective interventional trials to ensure recurrence risk in ctDNA-negative patients remains comparable to stage I disease.

During follow-up, ctDNA monitoring enabled early recurrence detection with 91.3% sensitivity and 94.2% specificity when using ctDNA-positive samples taken ≥14 days post-surgery. In selected cases, saliva samples provided a median lead time of 111 days over plasma. Patients who were ctDNA-positive post-treatment had a 36-fold increased risk of recurrence. This is highly relevant given the absence of guidelines recommending routine cross-sectional imaging during HNSCC follow-up and the lack of evidence that regular imaging improves relapse detection. The NCCN guidelines recommend early post-treatment imaging (within six months) for restaging but do not endorse routine, periodic whole-body or cross-sectional re-imaging for all asymptomatic patients beyond that unless there are symptoms or clinical concern^88^. European guidelines (EHNS/ESMO/ESTRO) similarly recommend imaging when clinically indicated (symptoms/abnormal exam) rather than routine scheduled imaging for all patients^2^. In practice, ctDNA monitoring could therefore provide an early and objective trigger for targeted investigations and timely salvage interventions where current modalities remain suboptimal. Plasma ctDNA profiling also helped differentiate lung metastases from SPTs, which are common in HNSCC survivors and difficult to distinguish histologically^7,58,59,89^.

As with all technologies, there are limitations that lead to challenges in the clinical applications. A key limitation is the requirement for very high sensitivity at very low eVAF typical of postoperative MRD, which is critical if ctDNA is to inform adjuvant treatment decisions. In our cohort, 13/23 patients who eventually recurred were ctDNA-negative immediately postoperatively (sample collected on day two to seven after surgery), suggesting a subset of recurrences may be missed with current approaches, particularly if only one postoperative timepoint is considered. Although the RaDaR assay used here was analytically validated to detect ctDNA down to parts-per-million (ppm) range in clinical studies^29,90^, and has reported high analytical sensitivity at 0.001% eVAF^37^, analytical limits do not always translate directly into required clinical sensitivity because of additional factors involved. These can include low shedding tumors, localized disease, and sampling timing. Small, slowly proliferating, or anatomically localized residual or recurrent disease with limited access to the vascular or lymphatic system may fall below the assay’s limit of detection at the time of sampling, which likely contributed to the subset of recurrences that were not ctDNA-positive immediately postoperatively in our cohort. Finally, timing matters, however, the most appropriate postoperative sampling time to detect MRD has not been defined yet for patients with HNSCC. In our cohort, sampling times were chosen between one and seven days, and relatively sparse sampling may reduce the chance of detecting transient ctDNA peaks at very low eVAF. Studies in other tumor entities have demonstrated that serial sampling can increase sensitivity, therefore reducing false negative rates^20,31,91^. It has been shown that surgical trauma can result in a four-week surge in wild-type cfDNA, which may dilute the ctDNA below assay detection limit^92^. It may therefore be beneficial to collect an additional sample four weeks postoperatively due to the potentially limited sensitivity of a single timepoint close to surgery. This may improve the overall NPV which is important for potential adjuvant treatment de-escalation plans in ctDNA-negative patients. The benefit that ctDNA-positive patients may have from adapted adjuvant therapy regimens will need to be determined in future prospective randomized clinical trials^91^. Similarly, during clinical follow-up, the sensitivity constraints necessitate serial sampling to increase detection probability and reduce false reassurance from isolated negative results. With these limitations in mind, ctDNA should currently be used as a complementary tool rather than a replacement for established clinical and radiological surveillance. Potential applications include ctDNA-stratified allocation of imaging resources for recurrence monitoring. For patients who become ctDNA-positive during clinical follow-up, this includes intensifying imaging immediately upon ctDNA detection, and, if a recurrence remains undetectable on CT imaging, potentially transitioning to alternative modalities, such as PET/CT^91^.

Whole genome cfDNA sequencing approaches may achieve even lower single-molecule or sub-ppm analytical sensitivity, suggesting the potential for improved detection of ultra-low-level MRD and early recurrence^19,93,94^. Non-tumor-informed methylation-based and fragmentomic approaches, or a combination of both (i.e. cfDNA fragmentomics-based deduction of epigenetic and transcriptomic information)^95^, may also offer complementary sensitivity for detecting MRD and recurrences^96–100^, particularly in low-shedding tumors but currently have different specificity-related limitations and are not yet validated for guiding therapy decisions, particularly in patients with HNSCC. Ultimately, sensitivity and specificity of ctDNA assays may vary depending on the clinical context in which they are applied and may require different thresholding strategies. During clinical surveillance, prioritizing detection of early recurrence may justify accepting a lower specificity, therefore allowing more low-level ctDNA detection considered to be positive, which in turn increases sensitivity for recurrence at the expense of more false positives. In contrast, for ctDNA-guided adjuvant treatment decisions, higher specificity thresholds are often required to avoid overtreatment.

In addition, a tumor-informed assay is not suitable to detect de-novo SPTs unless additional panels are generated from those lesions. Despite being the largest cohort of surgically treated patients with p16-negative HNSCC to date, our sample size remains small, limiting statistical power. Future studies would benefit from larger, more diverse cohorts and standardized ctDNA collection intervals. As most patients were p16-negative, findings may have limited applicability to p16-positive HNSCC. Tumor-informed assay design depends on sufficient tumor tissue for WES, which may not always be available, and requires a longer turnaround time to develop the panel and obtain results. However, tumor-agnostic assays may miss mutations in some patients due to HNSCC heterogeneity and scarcity of recurrent drivers^1,101–103^. Still, we demonstrate that diagnostic biopsy material is adequate for WES, potentially reducing turnaround time. Since ctDNA testing is not yet reimbursed in many countries, further interventional trials are needed before it becomes standard of care. Nonetheless, our findings support integrating ctDNA-based monitoring into future treatment strategies to improve outcomes in patients with HNSCC. With timely detection, we may be able to alter disease trajectory in ctDNA-positive patients before clinical relapse emerges.

## Methods

### Study design

LIONESS is a single-center, non-interventional, prospective experimental evidence-generating cohort study. Patients with HNSCC of the oral cavity, pharynx or larynx with stages I-IVb (AJCC 8^th^ edition of the UICC/AJCC staging system^46^), deemed resectable and scheduled for surgery were considered eligible. Patients with distant metastasis (cM1) or other active malignancies at the time of enrollment were excluded. Eighty-three patients were recruited at the Department of Otorhinolaryngology, Head and Neck Surgery at the LMU Klinikum in Munich, Germany, between April 2020 and September 2022, with a median follow-up of 29.5 months (range: 0–57 months). As suggested by the local multidisciplinary tumor board, patients received adjuvant (C)RT according to the National Comprehensive Cancer Network guidelines (NCCN Guidelines)^88^, if necessary. All patients were staged at diagnosis with CT and/or magnetic resonance imaging (MRI) to exclude distant metastasis. Immunohistochemical staining for p16 was done as part of the routine histopathological work-up. Follow-up has been conducted as part of the clinical routine. Pre- and postoperative blood and saliva sampling was performed in addition to routine clinical and laboratory examinations. During follow-up, cervical lymph node status was assessed by ultrasound imaging. CT and/or MRI was performed only in cases of detected abnormalities or if clinical symptoms were present^2^. In patients with small laryngeal or hypopharyngeal carcinomas who do not undergo adjuvant therapy, panendoscopy under general anesthesia is usually recommended by the tumor board at six to eight weeks postoperatively in order to detect early local recurrences. The primary objective was the identification of patients with MRD and early molecular-level recurrence during clinical follow-up with outcome measure determined as presence of ctDNA (i.e., presence of variants measured through RaDaR) in plasma and/or saliva of patients with locoregional HNSCC.

### Plasma sample collection and DNA extraction

Serial plasma samples were collected from 76 patients one to four days preoperatively as well as two to seven days postoperatively. Additional blood samples were obtained prior to and following adjuvant therapy, if indicated, as well as during follow-up visits. In case of a resectable recurrence, samples were obtained again pre- and postoperatively. Venous blood from study participants was collected by standard phlebotomy techniques in 2x10ml Cell-Free DNA blood collection tubes (Streck, La Vista, NE, USA) and sent for further processing to NeoGenomics Inc. (Fort Myers, Florida, USA). Plasma samples were prepared as soon as possible after collection and within seven days, with an initial centrifugation at 1600 g for 10 min and a second centrifugation of plasma aliquots at 20,000 g for 10 min. The buffy coat layer was separated during blood processing and stored at -80 °C. Control DNA was extracted from 200 µl of buffy coat (leukocytes) using QIAamp DNA Blood Mini Kit (Qiagen) and circulating cell-free DNA (cfDNA) was extracted using the QIAamp Circulating Nucleic Acid Kit (Qiagen). Buffy coat and cfDNA were quantified using digital PCR (BioRad QX200) as described previously^37^.

### Saliva sample collection and DNA extraction

Saliva samples were collected from 54 patients one to four days preoperatively as well as two to seven days postoperatively. Additional samples from selected patients were obtained prior to and following adjuvant therapy, if indicated. Saliva was passively collected by patients expectorating into a collection tube. When saliva collection was not feasible, patients were given 10 ml of aqua destillata or 0.9% sodium chloride solution to gargle for at least 30 s and subsequently transfer it into a collection tube. Saliva and mouth gargle samples were processed within two hours after collection by centrifugation at 2500 g for 20 minutes. The supernatant was stored at -20 °C until further processing. DNA was extracted using QIAamp Circulating Nucleic Acid Kit (Qiagen). cfDNA were quantified using digital PCR (BioRad QX200) as described previously^37^.

### Tumor whole exome sequencing

Tumor resection FFPE specimens of the resected specimen were obtained and ten 10 µm unstained slides and one hematoxylin and eosin-stained slide were cut from representative FFPE blocks. An experienced head and neck pathologist assessed tumor content and cellularity (median: 50%, range 40-70%), and suitable tumor areas were marked for macrodissection. Tumor tissue slides were sent for DNA extraction and WES to NeoGenomics Inc. (Fort Myers, Florida, USA). DNA was extracted from the provided FFPE tumor tissue samples using the Maxwell RSC DNA FFPE kits. The extracted DNA was processed with the KAPA Hyper Prep Kit and indexed uniquely. Resulting pre-capture libraries were quantified using the Quant-iT dsDNA High Sensitivity assay. Each library proceeded to exome enrichment and was analyzed on a fragment analyzer and quantified using the Quant-iT dsDNA High Sensitivity assay. Sequencing was performed on the HiSeq4000 platform (Illumina).

### Bioinformatics analysis for whole exome sequencing for patient-specific assay design

The analysis of tumor-only exome sequencing data was performed using a proprietary pipeline: samples were processed through fastq files processing, alignment, and variant calling. Germline variants were filtered out using custom filters that consider prior knowledge available from public single nucleotide polymorphism (SNP) databases while somatic variants were filtered based on multiple parameters including allele frequency and depth. Performance characteristics of the WES is shown in Supplementary Data 1 – File 2.

### RaDaR patient-specific assay design

RaDaR is a personalized ctDNA assay built on the InVision® platform, which utilizes multiplex PCR and targeted next generation sequencing (NGS)^104^. Somatic variants identified in the tumor tissue by WES were prioritized using proprietary algorithms to build a patient specific primer panel of up to 48 primer pairs capturing at least one somatic variant (Supplementary Data 1 – File 2). The personalized primer-panel was manufactured (IDT, Coralville, IA) and combined with a fixed primer panel of 21 common population-specific SNPs for quality control purposes during the NGS testing. An aliquot of the same tumor tissue DNA subjected to WES was used for panel qualification to confirm the accuracy and performance of the RaDaR panel design.

Following panel qualification, RaDaR assays were performed on plasma cfDNA alongside a buffy coat DNA control sample, which was used for identification and removal of germline variants and variants due to clonal hematopoiesis of indeterminate potential (CHIP) from the analysis, and as a positive amplification control. Multiplex PCR was performed with input concentrations ranging from 1452 to 20,000 amplifiable copies per sample, as measured by droplet digital PCR, with a median value of 14,550 copies.

### RaDaR sequencing analysis

RaDaR libraries were sequenced using the Nova-Seq 6000 system (Illumina Inc., San Diego, USA) and sequencing data analyzed in a multi-step process: fastq files were demultiplexed using *bcl2fastq*, reads were then aligned using the *bwa mem* alignment software and processed using proprietary software to identify primer pairs and count mutant and reference bases.

Individual variants were subject to QC in the process of calling residual disease positive or negative. Variants present in the buffy coat material or absent in the tumor tissue DNA were excluded from further analysis. Proprietary methods were used to call residual and recurrent disease: a statistical model was used to assess the statistical significance of the observed mutant counts for each variant and the information was integrated over the entire set of personalized variants to obtain evidence of tumor presence or absence at the sample level. This includes an assessment of the noise of each individual variant class and the sensitivity and specificity based on the number of variants in the panel. A sample will be called as positive for residual disease if its cumulative statistical score is above a pre-set threshold, as defined during analytical development. The tumor fraction estimated from this model was then reported (estimated variant allele frequency, eVAF). RaDaR’s analytical sensitivity at sample level has been previously shown to be 95% at 0.001% median eVAF^37^.

### Longitudinal plots of ctDNA detection levels and lead time analysis

Lead time was calculated as the interval between the first ctDNA-positive sample taken ≥14 days from surgery to the date of clinical confirmation of recurrence. Survival analysis was plotted using a Kaplan Meier curve.

### Statistical analysis

Statistical analysis of the measured and collected parameters was performed, unless stated otherwise, in R version 4.4.0.

#### Descriptive statistics

Descriptive analysis was performed with built-in packages and functions from The Comprehensive R Archive Network (CRAN) package tidyverse (version 2.0.0). Quantitative parameters were descriptively analyzed by calculating the minimum, median, harmonic mean, maximum values as well as standard deviation (SD), 1st and 3rd quartile and interquartile range (IQR) and visualized with histograms. Quantitative parameters were provided as mean ± SD. Differences between two groups of a quantitative parameter were visualized with boxplots. Qualitative parameters were shown as absolute and relative counts in frequency tables and visualized with bar plots. Differences of a qualitative parameter between two groups were analyzed with a contingency table with categories of the first parameter in columns and the second in rows and visualized with a stacked bar chart.

#### Univariate analysis

Inferential statistical analysis was performed with built-in packages and functions from the CRAN packages tidyverse and stats (version 4.4.0).

Analysis of the association between two qualitative, binary parameters with data represented in a contingency table was performed with Fisher’s exact test when at least one cell value was less than five. When this condition was not met, the Chi-squared test was used. To analyze the association between two qualitative, non-binary parameters with more than two groups to be compared, Pearson’s Chi-squared test was used.

For the analysis of the difference between the means of two independent groups of a quantitative parameter with a qualitative binary parameter defining the two groups, distribution of the quantitative data was first tested with the Kolmogorov-Smirnov test as well as a Q-Q plot. If the data of the two groups was normally distributed, the Student’s two-sample t-test was used. For a non-normal distribution, the non-parametric Mann-Whitney-U test was used.

Analysis of the difference between the means of three or more groups of a quantitative parameter with a qualitative parameter defining the groups was performed using the One-Way ANOVA test. Correlation analysis between two quantitative variables was performed using the Pearson Correlation Coefficient if the data was normally distributed. If this condition was not met, the Spearman Rank Correlation was used. All data tested in univariate analysis is deemed independent.

### Bioinformatics analysis of whole exome sequencing data for mutational analysis

For primary processing of WES data (alignment, variant calling, TMB calculation) the Illumina DRAGEN software v.4.3 was used (h38 reference genome). Variant annotation was conducted with openCRAVAT^105^. Two different filtering strategies were employed for generation of an oncoPrint. For the variants that were part of the custom personalized panel (panel variants), only germline variants were filtered out by (i) allele frequency in the Thousand Genomes Project (TGP) of < 0.01 and (ii) tumor allele frequency of < 0.3. For all non-panel variants additional filters were used with a focus on biological and clinical relevance: (iii) Sequence Ontology (“3_prime_UTR_variant”, “5_prime_UTR_variant”, “missense_variant”, “splice_site_variant”, “2kb_upstream_variant”, “2kb_downstream_variant”, “stop_gained”, “stop_lost”, and „start_lost“), (iv) exclusion ClinVar^106^ „benign“ variants”, and (v) exclusion of variants classified as „likely_benign“ by AlphaMissense algorithm^107^. The oncoPrint was plotted using the R package ComplexHeatmap. TMB between ctDNA high-shedders and low/non-shedders was compared by a Welch Two Sample t-test.

### RNA extraction from fresh frozen tumor samples

An 8 mm punch biopsy of a macroscopically vital area of the resected tumor specimen was collected intraoperatively. The retrieved specimen was covered with Ringer’s solution, immediately dried and embedded with Tissue-Tek O.C.T. Compound (Sakura Finetek, USA) for snap freezing in liquid nitrogen before being stored at -20 °C. Fresh frozen tumor tissue samples from *N* = 63 patients were processed for RNA extraction. 30 mg of tissue was homogenized using one stainless steel bead (7 mm mean diameter) with 600 µl of lysis buffer (Buffer RLT Plus, Qiagen, Germany) added, subsequently transferred to a TissueLyser (Qiagen, Germany) where disruption was performed in two cycles of 2 minutes each at 30 Hertz (Hz). Total RNA was extracted using RNeasy Mini Kit (Qiagen, Germany) according to manufacturer’s protocol instructions and stored in nuclease-free 1.5 ml microcentrifuge tube. For initial quality analysis, A260/A280 and A260/A320-ratios and the concentration for each sample were determined with the NanoPhotometer (Implen, Germany).

### 3’ RNA sequencing, preprocessing and quality analysis

The extracted total RNA samples were processed by Lexogen NGS Services (Vienna, Austria) for transcriptome sequencing. After quality analysis and library preparation, final 3’ RNA-seq was performed with 100 base-pair (bp) single-end mode on an Illumina HiSeq4000 Platform (Illumina, Inc., USA) by Lexogen.

#### Preprocessing

Demultiplexed FASTQ files with transcriptomic sequencing information for each sample were preprocessed as follows: FastQC (version 0.11.8) was used for the quality assessment of the raw reads. BBDUK (version 39.01) was then used for trimming adapters, polyA-tails, and bad quality reads prior to mapping to the human genome (GRCh38) using the STAR aligner (version 2.7.10b). Then HTSeq-count (version 2.0.3) in intersection-nonempty mode was used for counting the number of aligned reads that mapped to each gene (Ensembl gene IDs) of a sample. The counts per gene of each sample were imported into R and the individual counts were combined into a gene expression count matrix. The counts per Ensembl gene IDs were aggregated by sum for the corresponding HGNC gene symbols. We then filtered out all HGNC gene symbols with a mean expression across samples of less than 10 counts, which resulted in the final preprocessed gene expression matrix with genes in rows, samples in columns, and each cell value representing the counts of a gene in a sample.

#### Quality analysis

The total number of mapped reads per sample in million reads (mreads) was determined to assess quality of library preparation, sequencing quality, and alignment efficiency, and to identify outliers. The MT-RNA proportion of the total RNA was determined to assess the quality of the sequenced RNA. The total counts of mitochondrial genes, defined in Ensembl as “MT” and extracted from the gene expression matrix, were calculated for each sample and put into proportion with the total read count for that sample. The expression of the XIST-gene was used to identify a sample as male or female based on gene expression data. Values of the XIST-gene <5.0 in the gene expression matrix were considered to be male, >7.5 to be female and in between to be undefined computationally. These values were then compared with patient sex from the clinical annotation of the samples.

### Differential gene expression analysis, gene set enrichment analysis and pathway analysis

#### Differentially expressed genes (DEG)

Built-in packages and functions from the Bioconductor package DESeq2 (version 1.48.1) were used to compute DEG between samples with ctDNA detection (ctDNA shedders) and without detection (ctDNA low- or non-shedders) prior to surgery. Cut-off values of multiple-testing error-corrected p-values (false discovery rate (FDR)) using the Benjamini-Hochberg method ≤0.01 and of estimated absolute log2 fold change (|log2FC | ) of >1 were applied yielding the DEG between ctDNA shedders and low- or non-shedders.

#### Gene Set Enrichment Analysis (GSEA)

GSEA was performed using built-in packages and functions from the Bioconductor package clusterProfiler (version 4.12.0) and fgsea (version 1.30.0). All DEGs from the pairwise comparison of ctDNA shedders and low- or non-shedders with no cut-off values applied were ranked in decreasing order by the metric: sign of log2 fold change * negative decadic logarithm of p-value and used as input for GSEA analysis^108,109^. The cut-off for both p-value and for FDR-adjusted p-values was 0.05. Minimum gene size of gene sets in a collection was 10 genes and maximum was 500 genes. GSEA was performed on the Hallmarks gene set collection extracted from MSigDB. Results were visualized in a dotplot with Normalized Enrichment Score (NES) of the gene sets on the x-axis, FDR-adjusted p-value coded as color gradient and gene set size as dot size.

#### Pathway activity inference

Built-in packages and functions from the CRAN package Pathway RespOnsive GENes (PROGENy) (version 1.26.0) were used to estimate the activity of 14 signaling pathways. PROGENy pathway activity scores were calculated from the normalized gene expression count matrix resulting in a pathway activity matrix with samples in rows and the 14 pathways in columns. We used the top 500 genes of the PROGENy pathway signatures. The resulting matrix was visualized as a heatmap with samples annotated according to ctDNA status, with “Y” for ctDNA shedders and “N” for low- or non-shedders. We mean-centered the pathway activity scores per pathway to get z-scores, which were considered as Normalized Enrichment Scores (NES) that showed which pathways were activated or repressed in ctDNA shedders compared to low/non-shedders. Results were visualized in a histogram.

The genes that contributed most to the activation or repression of a pathway in ctDNA shedders compared to low- or non-shedders were visualized in a scatterplot. Responsiveness of a gene in a pathway was assessed as the intersection of its associated progeny weight in the pathway – on the x-axis in the scatterplot - and of its wald statistic value in differential gene expression on the y-axis of the scatterplot. The blue and red colors given to each gene represent the negative or positive contribution of a gene to the progeny pathway activity, respectively.

#### Transcription factor (TF) activity inference analysis

TF activity inference from the gene expression matrix was performed using the CollecTRI regulons collection containing a network of TF with their transcriptional targets and interactions weighted by mode of regulation, that can either be activation or inhibition^110^. The CollecTRI regulons were accessed through built-in packages and functions of the Bioconductor packages OmnipathR (version 3.11.10) and decoupleR (version 2.10.0). The Univariate Linear Model (ulm) method from the decoupleR package was then used with the CollecTRI regulons and the normalized gene expression matrix to fit a linear model for each gene and each TF. The t-value of the fitted slope was defined as the TF enrichment score which, if positive, was considered active or else negative.

To differentiate TF activities between ctDNA shedders and low- or non-shedders, the wald statistic from differential gene expression between ctDNA shedders and low- or non-shedders was extracted and used as input to the ulm method as described above. The top 12 TF activities both positive and negative were then selected to get the most activated and most repressed TF activities in ctDNA shedders compared to low- or non-shedders, respectively. These 24 TFs were visualized through their TF enrichment scores in a histogram centered around a score of 0.

For TF of interest, the genes contributing most to the activation or repression of the TF in ctDNA shedders compared to low- or non-shedders were visualized in a volcano plot where the contribution of a gene to TF activity was assessed as the intersection of the associated logfc value on the x-axis of the plot - and the negative decadic logarithm of the p-value on the y-axis of the plot. The blue and red colors represent the negative or positive contribution of a gene to the TF activity. If the sign of multiplying the mode of regulation (MOR) and t-value was negative, the contribution of a gene to TF activity was negative (blue), otherwise it was positive (red).

#### Virtual Inference of Protein-activity by Enriched Regulon (VIPER) analysis

Protein activity inference was calculated from the normalized gene expression matrix on a single-sample basis with built-in packages and functions from the Bioconductor package VIPER (version 1.38.0). The HNSCC regulon inferred by the ARACNe algorithm from HNSCC TCGA data needed for the analysis was retrieved in R with built-in packages and functions from the Bioconductor package arcane.networks (version 1.30.0). A protein activity matrix was computed using a Null model with regulatory proteins in rows, samples in columns and each value being an NES of a regulatory protein for a given sample.

To assess the difference in regulatory protein activity between ctDNA shedders and low- or non-shedders, the 10 most differentially activated proteins between ctDNA shedders and low- or non-shedders were identified using the Student’s t-test on the Protein Activity matrix.

### Immunohistochemistry (IHC) analysis

FFPE tumor blocks of the resected specimen (*N* = 71) were obtained and 10 µm unstained slides and one hematoxylin and eosin-stained slide were cut from representative FFPE blocks. Slides were stained for PD-L1 (Ventana SP263, ready to use), PD-1 (Ventana, NAT 105, ready to use), CD8 (Medac, C8/144B 1:50), CD3 (Zytomed SP7, 1:150) and Ki67 (Dako, MIB-1, 1:100) at the Institute of Pathology, LMU Munich, Germany. All IHC steps were performed in Ventana Benchmark Ultra (Ventana, USA) according to standard procedures from deparaffination to hematotoxylin counterstain. After quality analysis by an experienced pathologist, the following number of stained slides were used for downstream analysis: Ki67 (*N* = 32), PD-L1 (*N* = 64), PD-1 (*N* = 49), CD3 (*N* = 49), and CD8 (*N* = 49).

#### Image data acquisition and preprocessing using QuPath software

Slides were scanned with an Aperio GT 450 scanner (Leica Biosystems, Germany) at x40 magnification. For each biomarker, with exception of PD-L1, a project was created within QuPath (version 0.5.1-arm64), and the corresponding slides were imported into the respective project. Image type was set to *Brightfield (H-DAB)* and for each slide, the tumor area was annotated using QuPath. Stain vector and background estimates were determined using the *Estimate stain vectors* command in a representative region of the slide containing a background area as well as strong hematoxylin and 3,3’-Diaminobenzidine (DAB) staining and finetuned for optimal detection in subsequent analysis. This process was repeated for each slide before further analysis of CD3, CD8, Ki-67, and PD-1.

#### Analysis of CD3 and CD8 IHC with Immunoscore

After preprocessing of the slide images as described above, an experienced pathologist marked regions of the tumor core and the invasive margin in each slide manually. These were then annotated in QuPath as tumor core (CT) and invasive margin (IM) annotations. Quantification of CD3 and CD8 density in the slides was performed using the Positive cell count detection command on the CT and IM areas to identify individual CD3- respectively CD8-stained cells using the following parameters: Detection image – Optical density sum; Background radius: 5 µm with a median filter radius of 0; Sigma of 1.75 µm; Min. area of 8 µm^2^ and max. are of 500 µm^2^ with a cell expansion of 5 µm; Score compartment was set at Cell: DAB OD mean using the single threshold of 0.03 after finetuning for best possible detection. The density was calculated as average number of positive cells per mm^2^ from the number of positive cells and the detected area for the CT and IM areas. Visual verification of the results was performed on markup images showing the detected cells. Then, a batch script was generated which applied the above parameters and commands to all 49 slides. Positive cells per mm^2^ were then extracted as density scores. To determine the Immunoscore^111^, the median densities of CD3 in the tumor core (CD3-CT) and the invasive margin (CD3-IM) of a tumor were calculated. The same was done for CD8 (CD8-CT; CD8-IM). The median density of CD3-CT was 1501.06 positive cells/mm^2^, of CD3-IM 2402.43 positive cells/mm^2^, of CD8-CT 506.24 positive cells/mm^2^ and of CD8-IM 1010.36 positive cells/mm^2^. The density values of CD3 and CD8 in the CT and the IM were classified into “High” or “Low” depending on their value being above or below the median value in that area, respectively. A score of I0 for a tumor meant that CD3 and CD8 had a low density in the CT and the IM. I1 meant that one antigen (CD3 or CD8) had a high density in one location and so forth; I4 meant that CD3 and CD8 had high densities in the CT and IM. Fisher’s exact test was used for statistical analysis.

#### Analysis of PD-1 expression

To determine PD-1 expression, an experienced pathologist marked regions of the tumor core and the invasive margin in each slide manually. These were then annotated in QuPath as tumor core (CT) and invasive margin (IM) annotations as described previously. The percentage of positive cells out of all detected cells was taken and compared between ctDNA high-shedding (*N* = 48) and low- or non-shedding tumors (*N* = 5). Mann-Whitney U test was used for statistical analysis.

#### Analysis of PD-L1 IHC with Combined positive Score (CPS)

Quantification of PD-L1 density in the slides was performed manually by an experienced pathologist. CPS were determined as quantification measures for each slide. Mann-Whitney-U test was used for statistical analysis.

#### Analysis of Ki67 IHC with proliferation index

To determine the Ki67 proliferation index, an experienced pathologist marked the tumor region in each slide manually, which was then annotated in QuPath. H-DAB values were set as explained above and positive cell detection was run with other parameter values after finetuning. The parameter values that were different from those in CD3 and CD8 analysis were: Detection image – Hematoxylin OD; Background radius: 8 µm with a median filter radius of 0.5; Sigma of 2.0 µm. Score compartment was set at Nucleus: DAB OD mean as Ki67 is a nuclear marker with a threshold of 0.08. A batch script was generated, and the positive cell detection was performed on all slides. The percentage of positive cells was then extracted and used as the Ki67 proliferation index. Mann-Whitney-U test was used for statistical analysis.

### Ethics approval and consent to participate

Written-informed consent was obtained from all patients prior to their participation in the LIONESS study, which was approved by the local ethics committee of the LMU Klinikum in Munich, Germany, (ref. no. 18-446) as well as the data protection officer. Study participants consented to sharing pseudonymized data and samples within and outside of the European Union. The study has been conducted in accordance with the Declaration of Helsinki and in keeping with the rules of good clinical practice and according to the German laws and ethical standards.

## Supplementary information

Supplementary Figures

Supplementary Data 1

## Data Availability

The sequencing data generated and analyzed in this study have been deposited in the European Genome-phenome Archive (EGA) under accession number EGAD50000001487. Access to the data is controlled and can be obtained following EGA’s standard application procedures.
