# Supplementary material for: Personalized ctDNA analysis for detection of residual disease and recurrence in surgically treated HNSCC patients
Source: NPJ Precis Oncol. 2026 Feb 3;10:103. doi: 10.1038/s41698-026-01309-0 (PMC12966284; doi:10.1038/s41698-026-01309-0)
Supplement: Supplementary file 1 — Supplementary Figures [file 41698_2026_1309_MOESM1_ESM.pdf]

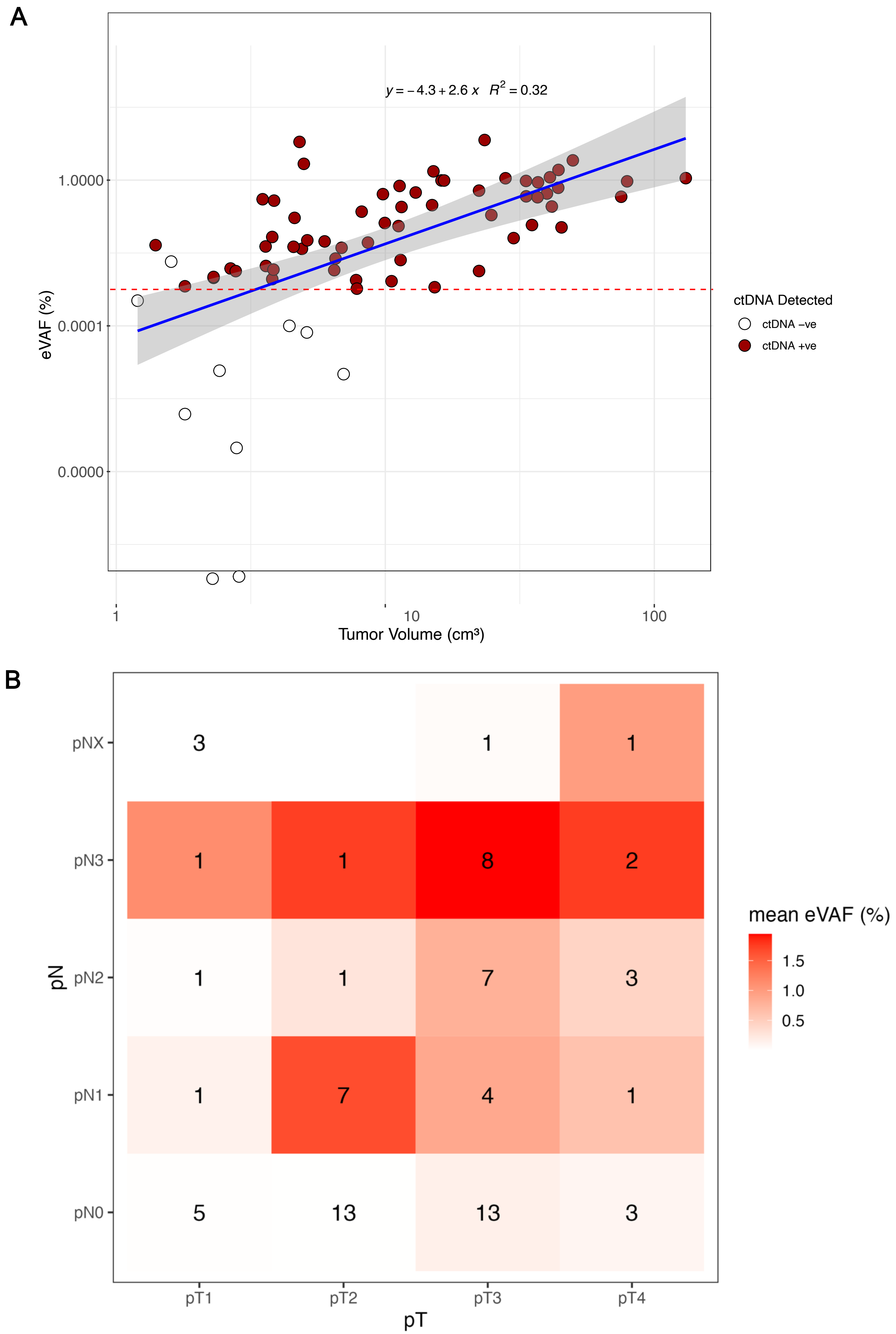

Supplementary Figure 1: Comparison of preoperative ctDNA detection with tumor characteristics.
A, Linear regression analysis between preoperative tumor volumes from staging CT scans and preoperative plasma ctDNA % eVAF levels. Best-fit regression is indicated by a blue solid line, red circles indicate ctDNA-positive samples and white circles ctDNA-negative samples. **B**, Heatmap of the mean of the raw % eVAF for each pathological tumor/nodal stage group. CT, computed tomography; eVAF, estimated variant allele frequency.

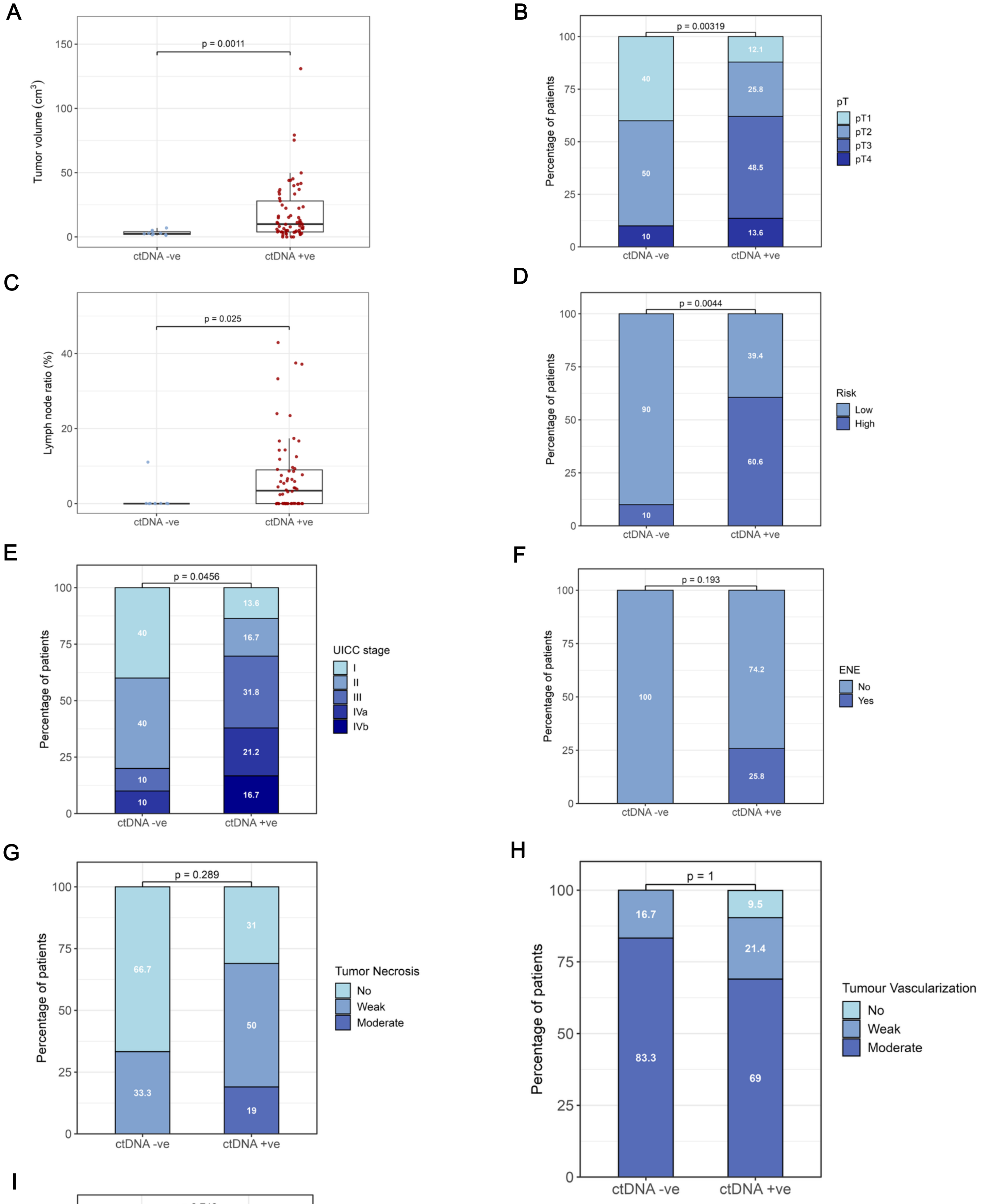

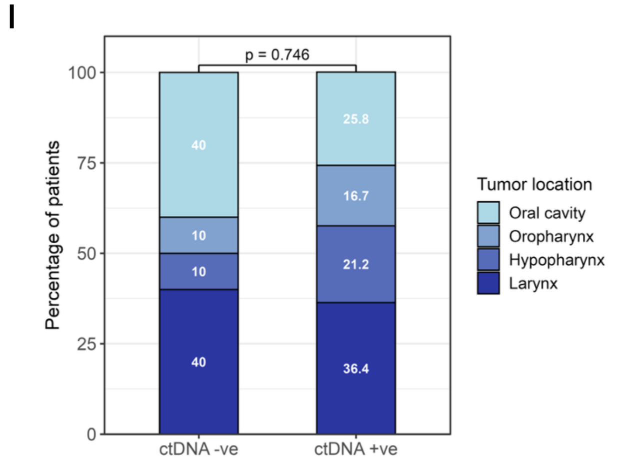

Supplementary Figure 2: Comparison of preoperative ctDNA detection with clinical and histopathological characteristics.
Comparison of patients with and without preoperative ctDNA-detection based on tumor volumes from staging CT scans (**A**), pathological tumor stage (**B**), lymph node ratio (**C**), histopathological risk group; high-risk encompassing L1, V1, Pn1, positive nodal stage or ENE-positivity (**D**), prognostic UICC stage group (**E**), ENE (**F**), tumor necrosis as determined by CT staging scans (**G**), tumor vascularity as determined by CT staging scans (**H**), and localization of the primary tumor (**I**). L1, lymphatic invasion; V1, venous invasion; Pn1, perineural invasion; ENE, extranodal extension; CT, computed tomography.

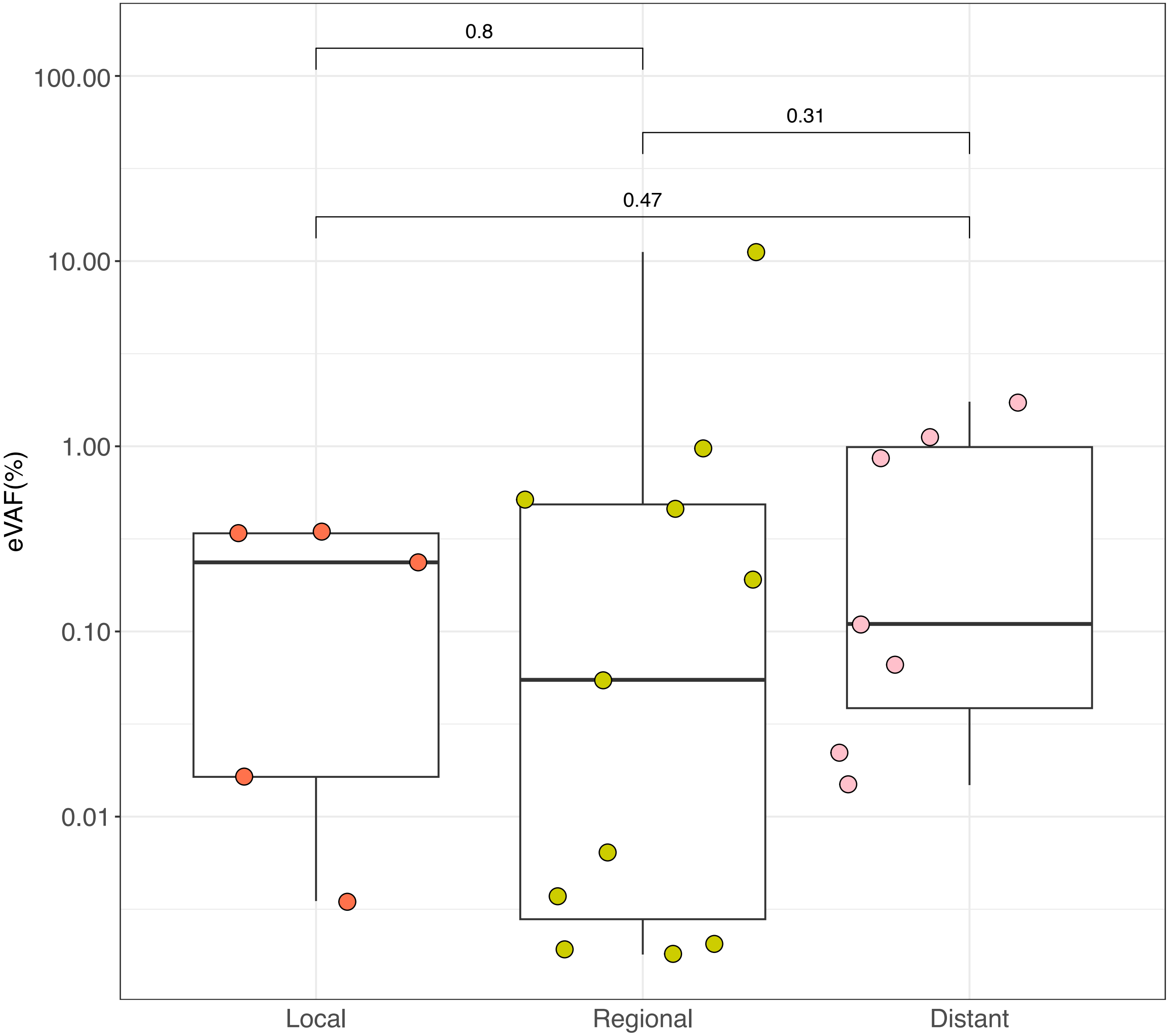

Supplementary Figure 3: Comparison of preoperative ctDNA % eVAF detection levels with type of recurrence. Boxplot center line indicates the median, box limits indicate the upper and lower quartiles.

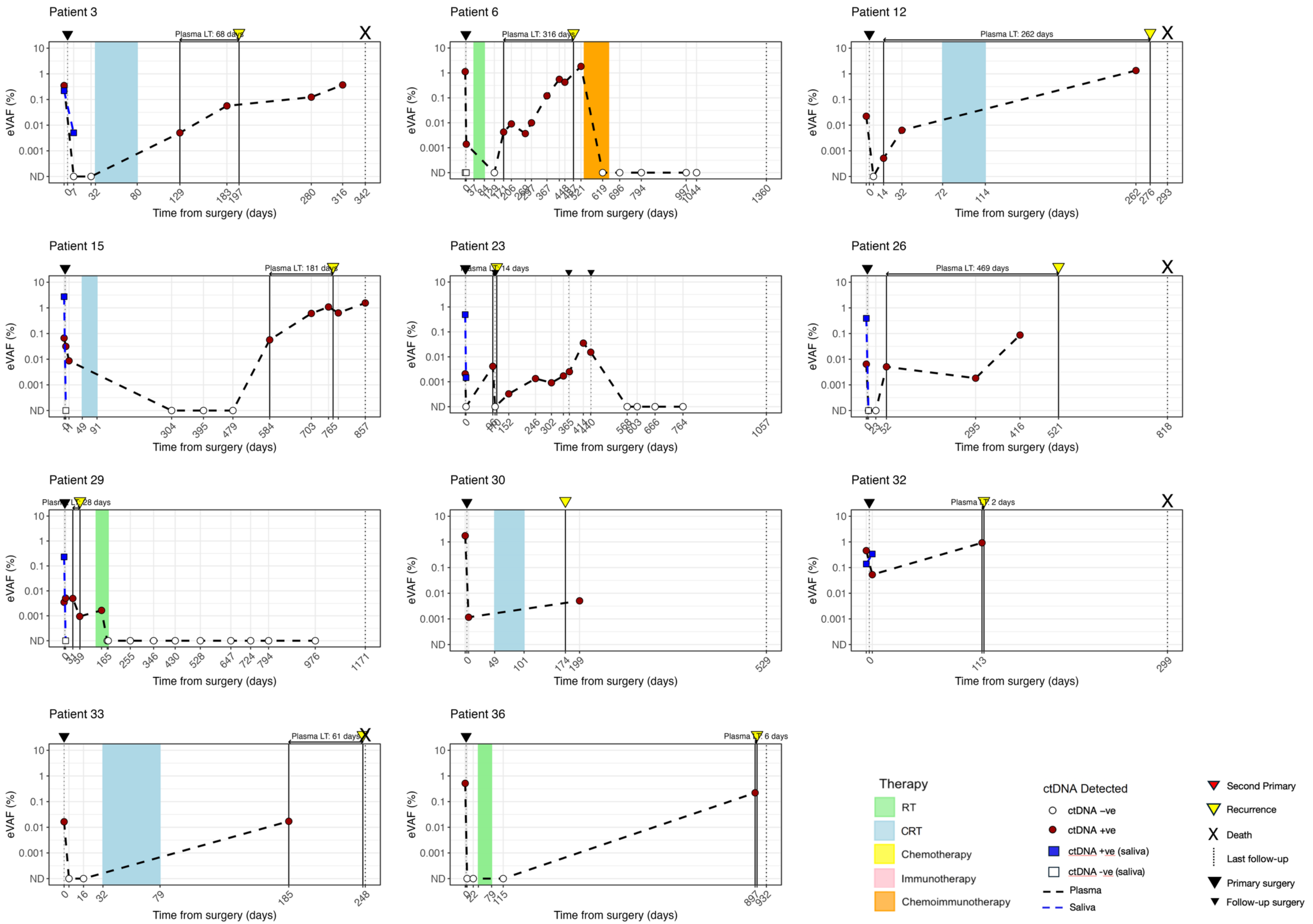
Patient 3
Plasma LT: 68 days
Patient 6
Plasma LT: 316 days
Patient 12
Plasma LT: 262 days
Patient 15
Plasma LT: 181 days
Patient 23
Patient 26
Plasma LT: 469 days
Patient 29
Patient 30
Patient 32
Patient 33
Plasma LT: 61 days
Patient 36
eVAF (%)
Time from surgery (days)
Therapy
RT
CRT
Chemotherapy
Immunotherapy
Chemoimmunotherapy
ctDNA Detected
ctDNA −ve
ctDNA +ve
ctDNA +ve (saliva)
ctDNA -ve (saliva)
Plasma
Saliva
Second Primary
Recurrence
Death
Last follow-up
Primary surgery
Follow-up surgery

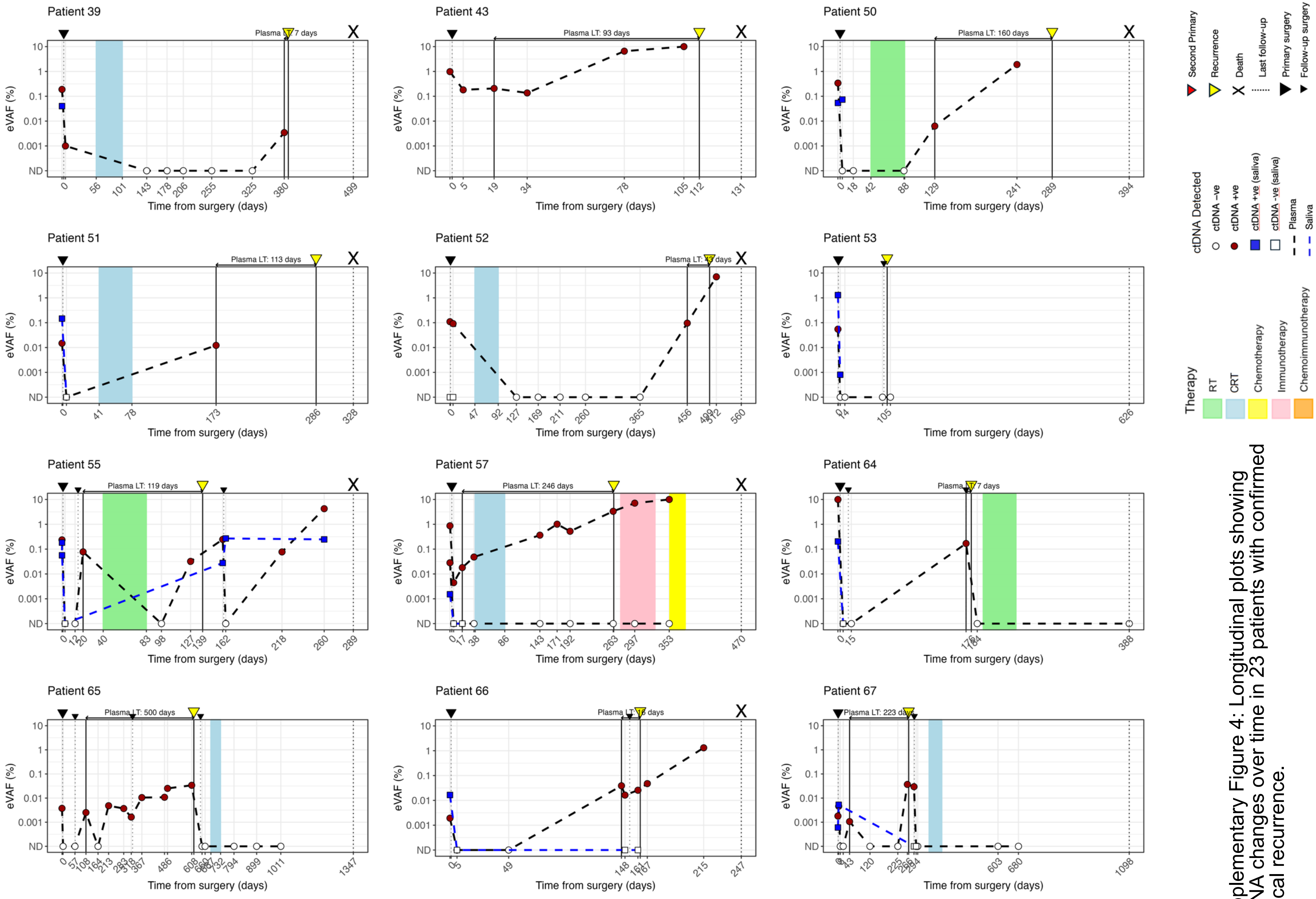

Supplementary Figure 4: Longitudinal plots showing ctDNA changes over time in 23 patients with confirmed clinical recurrence.

A

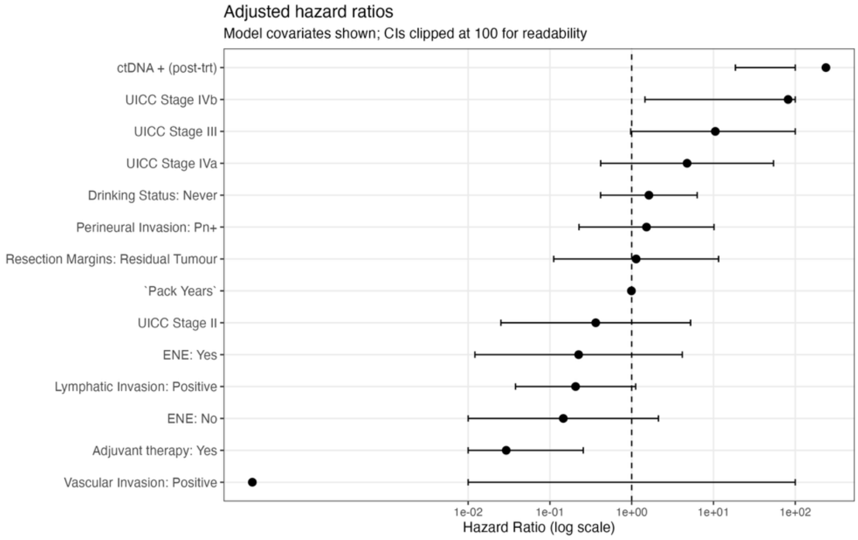

Term	HR (95% CI)	P
N (events)	71 (21)	
Concordance (se)	0.916 (0.024)	
LR test p	6.44e-08	
Wald test p	6.64e-02	
Score test p	4.04e-08	
Vascular Invasion: Positive	0.00 (0.00–Inf)	0.999
Adjuvant therapy: Yes	0.03 (0.00–0.26)	0.001
ENE: No	0.15 (0.01–2.12)	0.159
Lymphatic Invasion: Positive	0.21 (0.04–1.12)	0.067
ENE: Yes	0.22 (0.01–4.16)	0.316
UICC Stage II	0.36 (0.03–5.25)	0.458
`Pack Years`	0.99 (0.96–1.02)	0.654
Resection Margins: Residual Tumour	1.13 (0.11–11.55)	0.916
Perineural Invasion: Pn+	1.52 (0.23–10.15)	0.667
Drinking Status: Never	1.62 (0.42–6.33)	0.485
UICC Stage IVa	4.76 (0.42–54.25)	0.209
UICC Stage III	10.53 (0.97–114.19)	0.053
UICC Stage IVb	81.80 (1.45–4608.93)	0.032
ctDNA + (post-trt)	237.28 (18.55–3035.78)	<0.001

B

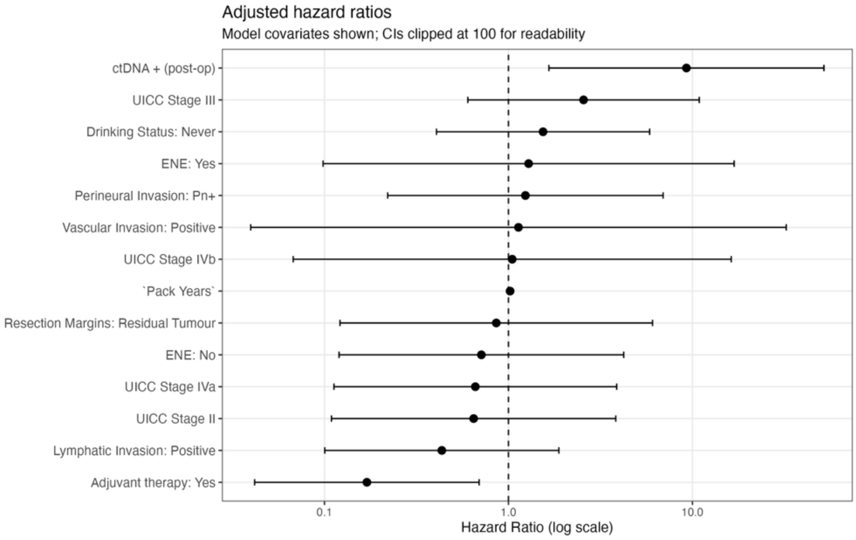

Term	HR (95% CI)	P
N (events)	71 (21)	
Concordance (se)	0.916 (0.024)	
LR test p	6.44e-08	
Wald test p	6.64e-02	
Score test p	4.04e-08	
Adjuvant therapy: Yes	0.17 (0.04–0.69)	0.013
Lymphatic Invasion: Positive	0.43 (0.10–1.88)	0.265
UICC Stage II	0.65 (0.11–3.83)	0.631
UICC Stage IVa	0.66 (0.11–3.88)	0.646
ENE: No	0.71 (0.12–4.23)	0.709
Resection Margins: Residual Tumour	0.86 (0.12–6.07)	0.879
`Pack Years`	1.02 (1.00–1.04)	0.053
UICC Stage IVb	1.05 (0.07–16.25)	0.973
Vascular Invasion: Positive	1.13 (0.04–32.35)	0.942
Perineural Invasion: Pn+	1.24 (0.22–6.93)	0.809
ENE: Yes	1.29 (0.10–16.85)	0.848
Drinking Status: Never	1.54 (0.41–5.85)	0.525
UICC Stage III	2.56 (0.60–10.89)	0.203
ctDNA + (post-op)	9.28 (1.66–51.86)	0.011

Supplementary Figure 5: Multivariable Cox proportional hazards model of post-treatment ctDNA positivity on RFS (**A**) and of postoperative ctDNA positivity on RFS (**B**). RFS, recurrence-free survival.

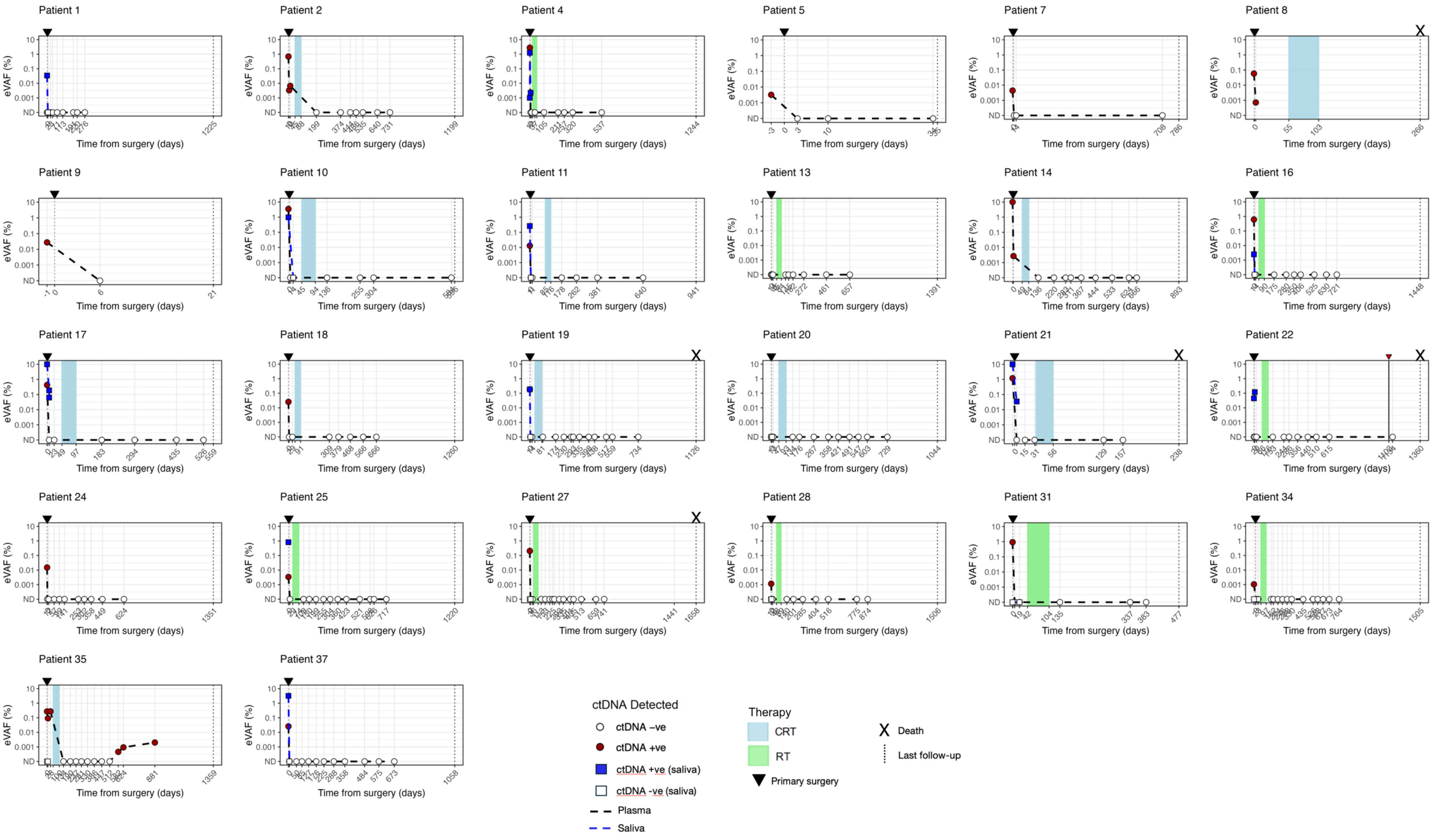
Patient 1
Patient 2
Patient 4
Patient 5
Patient 7
Patient 8
Patient 9
Patient 10
Patient 11
Patient 13
Patient 14
Patient 16
Patient 17
Patient 18
Patient 19
Patient 20
Patient 21
Patient 22
Patient 24
Patient 25
Patient 27
Patient 28
Patient 31
Patient 34
Patient 35
Patient 37
eVAF (%)
Time from surgery (days)
ctDNA Detected
ctDNA –ve
ctDNA +ve
ctDNA +ve (saliva)
ctDNA -ve (saliva)
Plasma
Saliva
Therapy
CRT
RT
Primary surgery
X Death
Last follow-up

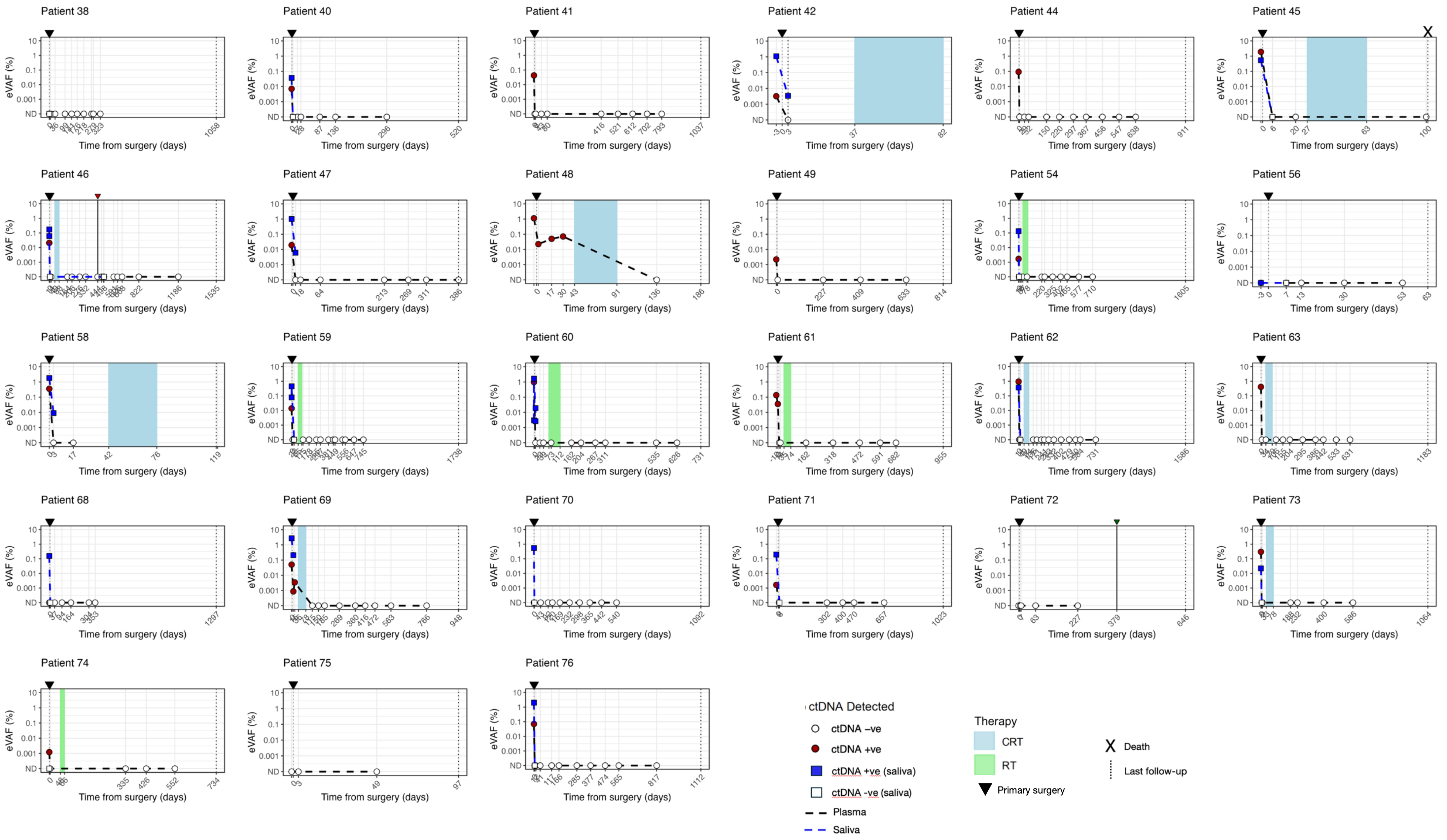

Supplementary Figure 6: Longitudinal plots showing ctDNA changes over time in 53 patients without clinical recurrence.

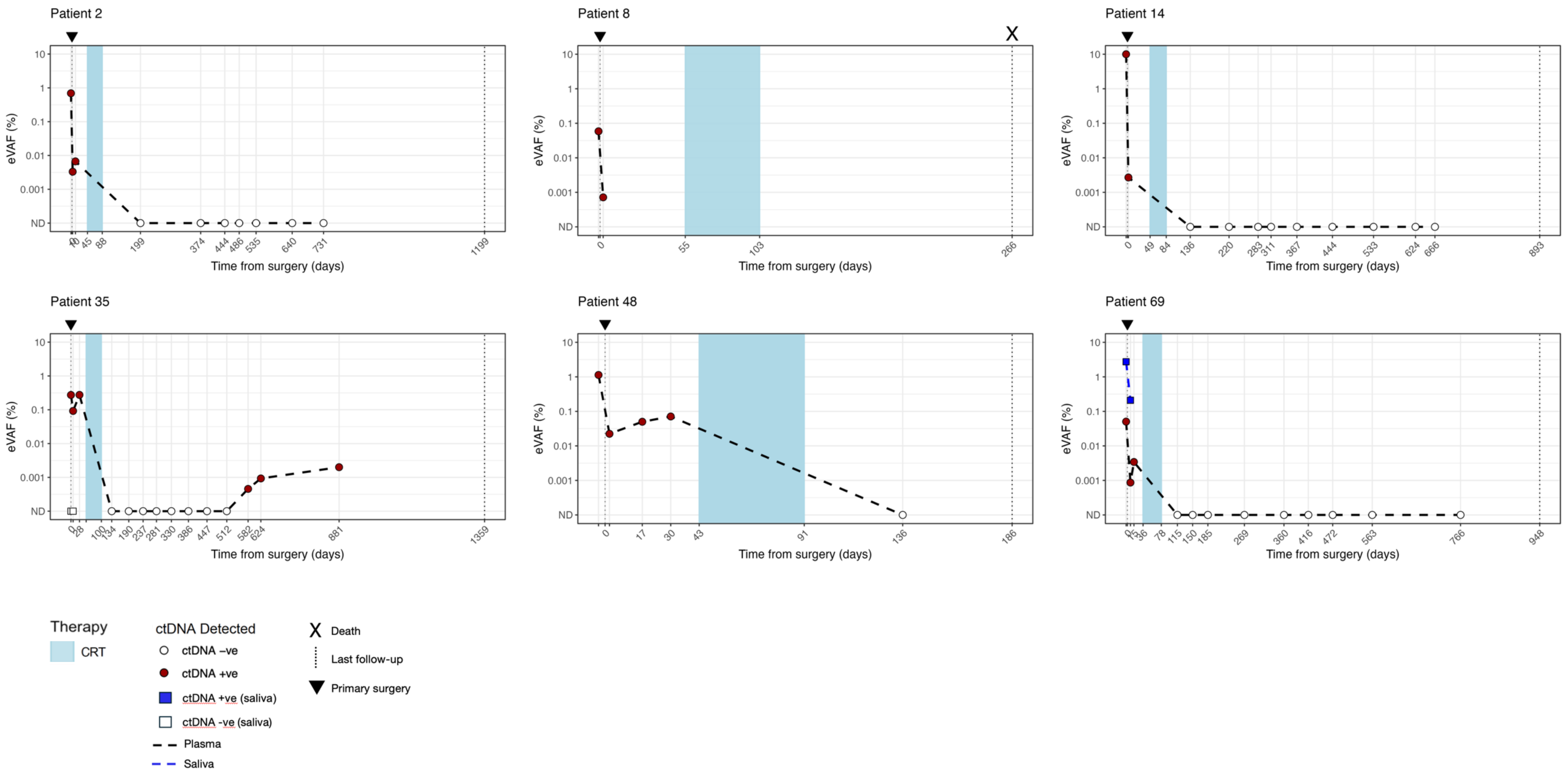

Supplementary Figure 7: Longitudinal ctDNA profiles in six MRD-positive patients without clinical evidence of recurrence, all of whom received adjuvant chemoradiotherapy. Red circles indicate ctDNA-positive samples and white circles ctDNA-negative samples. With exception of one patient (8) with no post-treatment samples available for testing, all remaining patients experienced ctDNA clearance following end of adjuvant therapy. MRD, molecular residual disease.

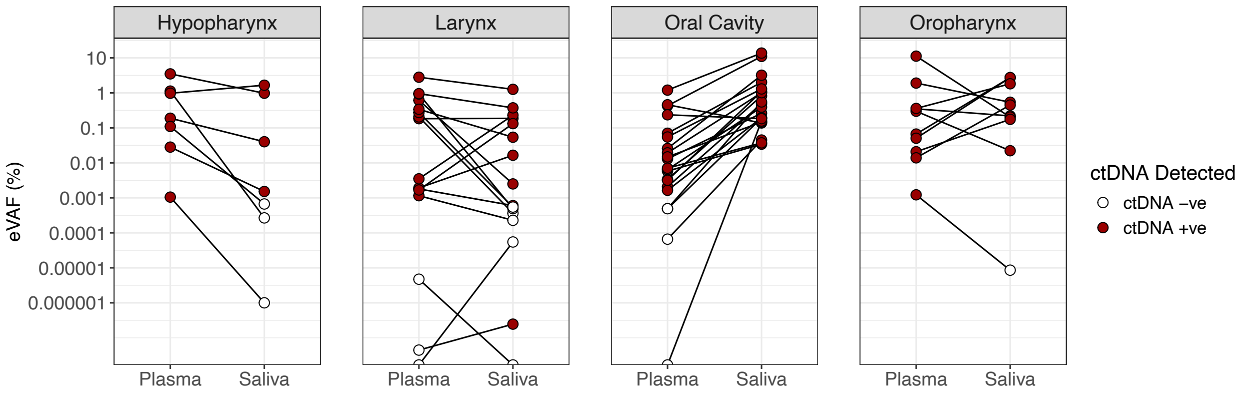

Site	Patients	Concordance (%)	Plasma +ve	Saliva +ve	%Difference (Plasma vs. Saliva)
OC	21	16/21 (76%)	16/21	21/21	76% vs. 100%
OP	10	9/10 (90%)	10/10	9/10	100% vs. 90%
HP	7	4/7 (57%)	7/7	4/7	100% vs. 57%
L	16	11/16 (69%)	13/16	10/16	81% vs. 62%
**Total**	54	40/54 (74%)	46/54 (85%)	44/54 (81%)	

Supplementary Figure 8: Comparison of ctDNA detection in plasma and saliva for different anatomical localizations.
Preoperative % eVAF for plasma and matched saliva samples subdivided into anatomical localizations. Table showing concordance rates between preoperative saliva and corresponding plasma samples for different anatomical localizations. P-values <0.05 are considered as statistically significant.
eVAF, estimated variant allele frequency; OC, oral cavity; OP, oropharynx; HP, hypopharynx; L, larynx.

A

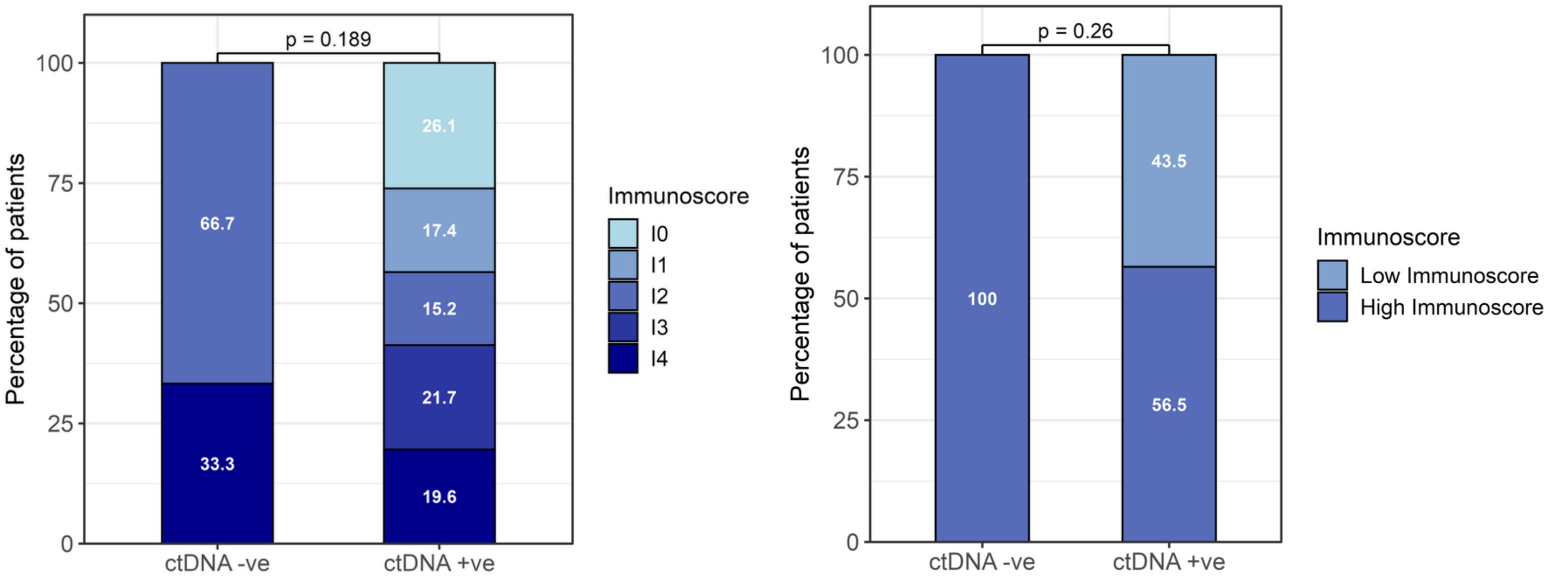

Preoperative ctDNA detection	I0	I1	I2	I3	I4	Total
**ctDNA -ve**	0	0	2	0	1	3
**ctDNA +ve**	12	8	7	10	9	46
**Total**	12	8	9	10	10	49

Preoperative ctDNA detection	Low Immunoscore	High Immunoscore	Total
**ctDNA -ve**	0	3	3
**ctDNA +ve**	20	26	46
**Total**	20	29	49

B

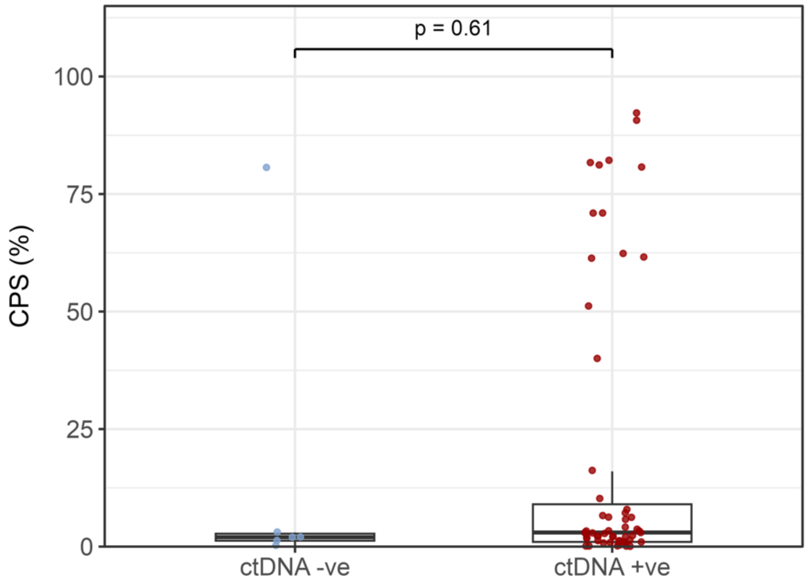

Preoperative ctDNA detection	Min.	Median	Mean	Max.	SD	Q1	Q3	IQR
**ctDNA -ve**	0.0	2.0	14.8	81.0	32.4	1.3	2.8	1.5
**ctDNA +ve**	0.0	3.0	17.8	92.0	29.7	1.0	9.0	8.0

Supplementary Figure 9: Comparison of immunohistochemical characteristics between ctDNA high-shedders vs. low/non-shedders.
A, Comparison of patients with and without preoperative ctDNA detection according to CD3/CD8 immunoscores. CD3 and CD8 densities were assessed in the tumor core and invasive margin, and categorized as "High" or "Low" based on whether their values were above or below the median for each region. Immunoscores ranged from I0 to I4: I0 indicates low CD3 and CD8 densities in both regions; I1–I3 reflect increasing combinations of high marker density in one or more locations; I4 indicates high CD3 and CD8 densities in both the tumor core and invasive margin. P-value from Fisher’s exact test. **B**, Boxplot comparing patients with and without preoperative ctDNA-detection based on CPS for PD-L1 expression. P-value from Mann-Whitney U test. Boxplot center line indicates the median, box limits indicate the upper and lower quartiles. CPS, combined positive score.

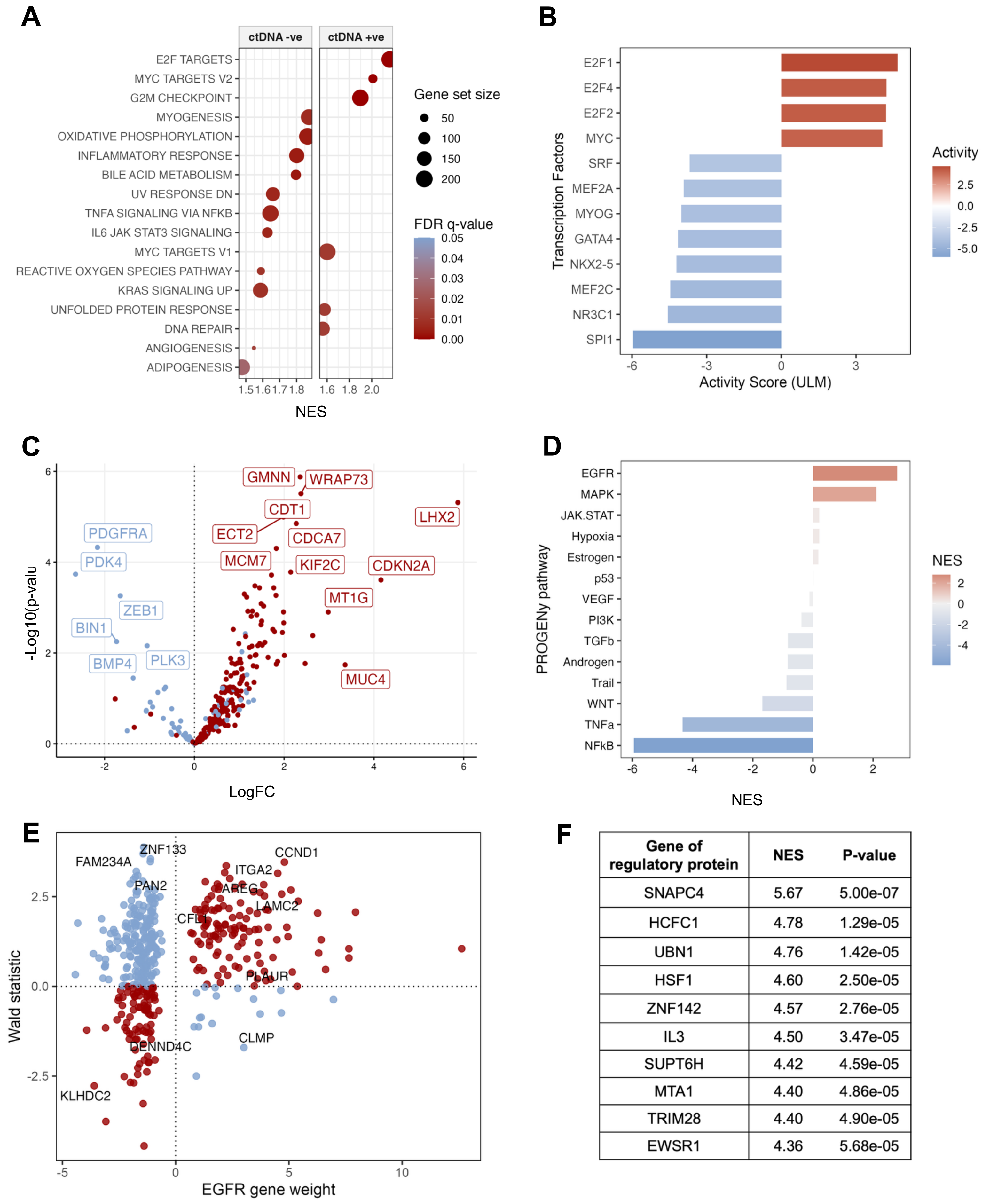

Gene of regulatory protein	NES	P-value
SNAPC4	5.67	5.00e-07
HCFC1	4.78	1.29e-05
UBN1	4.76	1.42e-05
HSF1	4.60	2.50e-05
ZNF142	4.57	2.76e-05
IL3	4.50	3.47e-05
SUPT6H	4.42	4.59e-05
MTA1	4.40	4.86e-05
TRIM28	4.40	4.90e-05
EWSR1	4.36	5.68e-05

Supplementary Figure 10: Comparison of transcriptomic characteristics between ctDNA high-shedders vs. low/non-shedders. **A**, GSEA (phenotype mode) of Hallmark collection of MSigDB containing 50 cancer-related gene sets. NES for significantly (adjusted p-value<0.05 and FDR<0.05) up- (NES>0) and downregulated (NES<0) gene sets are shown for ctDNA low/non-shedders (ctDNA -ve) vs. high-shedders (ctDNA +ve). **B**, Changes in TF activity in ctDNA low/non-shedders vs. high-shedders. **C**, Most differentially expressed target genes of the TF E2F1. Genes contributing most to the activation or repression of E2F1 in ctDNA low/non-shedders compared to high-shedders were visualized in a volcano plot. The blue and red colors represent the negative or positive contribution of a gene to the TF activity, respectively. **D**, NES of pathways based on PROGENy activity scores. **E**, Most responsive genes in the EGFR pathway are given by wald statistic for differential expression between ctDNA low/non-shedders and high-shedders and weight of the gene in the PROGENy pathway. **F**, Ten most differentially expressed regulatory proteins as calculated by VIPER algorithm that infers a protein activity matrix for each sample from gene expression data. GSEA, Gene Set Enrichment

Supplementary Data 1: Summary of clinical data, sequencing data as well as ctDNA assay performance data.
